# Modeling SARS-CoV-2 viral kinetics and association with mortality in hospitalized patients from the French COVID cohort

**DOI:** 10.1073/pnas.2017962118

**Published:** 2021-02-03

**Authors:** Nadège Néant, Guillaume Lingas, Quentin Le Hingrat, Jade Ghosn, Ilka Engelmann, Quentin Lepiller, Alexandre Gaymard, Virginie Ferré, Cédric Hartard, Jean-Christophe Plantier, Vincent Thibault, Julien Marlet, Brigitte Montes, Kevin Bouiller, François-Xavier Lescure, Jean-François Timsit, Emmanuel Faure, Julien Poissy, Christian Chidiac, François Raffi, Antoine Kimmoun, Manuel Etienne, Jean-Christophe Richard, Pierre Tattevin, Denis Garot, Vincent Le Moing, Delphine Bachelet, Coralie Tardivon, Xavier Duval, Yazdan Yazdanpanah, France Mentré, Cédric Laouénan, Benoit Visseaux, Jérémie Guedj, for the French COVID Cohort Investigators and French Cohort Study groups

**Affiliations:** ^a^Université de Paris, INSERM, IAME, F-75018 Paris, France;; ^b^AP-HP, Hôpital Bichat, Laboratoire de Virologie, F-75018 Paris, France;; ^c^AP-HP, Hopital Bichat, Service de Maladies Infectieuses et Tropicales, F-75018 Paris, France;; ^d^Univ. Lille, Virology Laboratory, EA3610, Institute of Microbiology, Centre Hospitalier-Universitaire de Lille, F-59037 Lille Cedex, France;; ^e^Laboratoire de Virologie, Centre Hospitalier-Universitaire de Besançon, F-25000 Besançon, France;; ^f^Laboratoire de Virologie, Institut des Agents Infectieux, Hospices Civils de Lyon, Groupement Hospitalier Nord, F-69004 Lyon, France;; ^g^Centre National de Référence des Virus Respiratoires, Hospices Civils de Lyon, Groupement Hospitalier Nord, F-69004 Lyon, France;; ^h^Service de Virologie, Centre Hospitalier-Universitaire de Nantes, F-44093 Nantes, France;; ^i^Laboratoire de Microbiologie, Centre Hospitalier-Universitaire de Nancy, F-54000 Nancy, France;; ^j^Université de Lorraine, CNRS, Laboratoire de Chimie Physique et Microbiologie pour les Matériaux et l'Environnement, F-54000 Nancy, France;; ^k^Normandie University, UNIROUEN Rouen, EA2656, Virology, Rouen University Hospital, F-76000 Rouen, France;; ^l^Virology, Pontchaillou University Hospital, F-35033 Rennes cedex, France;; ^m^Laboratoire de Virologie, Centre Hospitalier-Universitaire de Bretonneau, F-37044 Tours, France;; ^n^INSERM UMR 1259, Université de Tours, F-37044 Tours, France;; ^o^Laboratoire de Virologie, Centre Hospitalier-Universitaire de Montpellier, F-34295 Montpellier, France;; ^p^Infectious and Tropical Disease Department, Besancon University Hospital, F-25000 Besancon, France;; ^q^UMR CNRS 6249, Chrono Environnement, University of Bourgogne Franche-Comté, F-25000 Besancon, France;; ^r^AP-HP, Hôpital Bichat, Service de Réanimation Médicale et Infectieuse, F-75018 Paris, France;; ^s^Centre Hospitalier-Universitaire de Lille, Univ. Lille, Infectious Disease Department, CNRS, Inserm, U1019-UMR9017-CIIL, F-59000 Lille, France;; ^t^Université de Lille, INSERM U1285, Centre Hospitalier-Universitaire de Lille, Pôle de réanimation, CNRS, UMR 8576-UGSF-Unité de Glycobiologie Structurale et Fonctionnelle, F-59000 Lille, France;; ^u^Infectious and Tropical Disease Department, Croix-Rousse Hospital, University Hospital of Lyon, F-69004 Lyon, France;; ^v^Service de Maladies Infectieuses et Tropicales, Centre Hospitalier-Universitaire de Nantes, F-44093 Nantes, France;; ^w^Centre d'Investigation Clinique Unité d'Investigation Clinique 1413 INSERM, Centre Hospitalier-Universitaire de Nantes, F-44093 Nantes, France;; ^x^Université de Lorraine, Centre Hospitalier Régional Universitaire de Nancy, INSERM U1116, F-CRIN INICRCT, Service de Médecine Intensive et Réanimation Brabois, F-54000 Nancy, France;; ^y^Infectious Diseases Department, Rouen University Hospital, F-76000 Rouen, France;; ^z^Lyon University, CREATIS, CNRS UMR5220, INSERM U1044, INSA, F-69000 Lyon, France;; ^aa^Intensive Care Unit, Hospices Civils de Lyon, F-69002 Lyon, France;; ^bb^Infectious Diseases and Intensive Care Unit, Pontchaillou University Hospital, F-35000 Rennes, France;; ^cc^Centre Hospitalier Régional Universitaire de Tours, Service de Médecine Intensive Réanimation, F-37044 Tours Cedex 9, France;; ^dd^Tropical and Infectious Diseases, Saint Eloi Hospital, Université de Montpellier, Medical School, Montpellier University Hospital, F-34295 Montpellier Cedex 5, France;; ^ee^AP-HP, Hôpital Bichat, Department of Epidemiology Biostatistics and Clinical Research, F-75018 Paris, France;; ^ff^AP-HP, Hôpital Bichat, Centre d'Investigation Clinique, INSERM CIC-1425, F-75018 Paris, France

**Keywords:** SARS-CoV-2, viral dynamics, mortality

## Abstract

The characterization of severe acute respiratory syndrome coronavirus 2 (SARS-CoV-2) viral kinetics in hospitalized patients and its association with mortality is unknown. We analyzed death and nasopharyngeal viral kinetics in 655 hospitalized patients from the prospective French COVID cohort. The model predicted a median peak viral load that coincided with symptom onset. Patients with age ≥65 y had a smaller loss rate of infected cells, leading to a delayed median time to viral clearance occurring 16 d after symptom onset as compared to 13 d in younger patients (*P* < 10^−4^). In multivariate analysis, the risk factors associated with mortality were age ≥65 y, male gender, and presence of chronic pulmonary disease (hazard ratio [HR] > 2.0). Using a joint model, viral dynamics after hospital admission was an independent predictor of mortality (HR = 1.31, *P* < 10^−3^). Finally, we used our model to simulate the effects of effective pharmacological interventions on time to viral clearance and mortality. A treatment able to reduce viral production by 90% upon hospital admission would shorten the time to viral clearance by 2.0 and 2.9 d in patients of age <65 y and ≥65 y, respectively. Assuming that the association between viral dynamics and mortality would remain similar to that observed in our population, this could translate into a reduction of mortality from 19 to 14% in patients of age ≥65 y with risk factors. Our results show that viral dynamics is associated with mortality in hospitalized patients. Strategies aiming to reduce viral load could have an effect on mortality rate in this population.

## Significance

A detailed characterization of viral load kinetics and its association with disease evolution is key to understand the virus pathogenesis, identify high-risk patients, and design better treatment strategies. We here analyze the mortality and the virological information collected in 655 hospitalized patients, including 284 with longitudinal measurements, and we build a mathematical model of virus dynamics and survival. We predict that peak viral load occurs 1 d before symptom onset, on average, and that dynamics of decline after peak is slower in older patients. Viral load dynamics after hospital admission is an independent predictor of the risk of death, suggesting that prolonged viral shedding of high quantities of virus is associated with poor outcome in this population.

The severe acute respiratory syndrome coronavirus 2 (SARS-CoV-2) which originated in Wuhan, China, at the end of December 2019, has spread rapidly around the world, resulting, in November 2020, in over 60 million confirmed cases and more than 1.4 million deaths worldwide (1). Dozens of studies have evaluated risk factors or comorbidities associated with death (2–7), in particular, male gender, older age, diabetes, severe asthma, obesity, or chronic kidney diseases (7).

In other acute or chronic viral diseases (influenza, HIV, hepatitis C virus in particular), the characterization of viral load kinetics has played an important role in understanding the pathogenesis of the virus, identifying most at risks patients, and designing antiviral drugs (8–11). In the case of SARS-CoV-2, viral kinetics remain poorly characterized, and its association with disease evolution is controversial. This is due to the fact that many studies rely either on large cross-sectional analyses with few patients having serial data points or, conversely, on detailed but small series of patients, often with a mild disease (12, 13). Moreover, the discrepancies in the time of samplings, the definition of disease severity, and the populations analyzed make it difficult to get a clear picture of viral kinetics. Despite these heterogeneous data, pieces of evidence accumulate to suggest that the incubation period is about 5 d, and that the peak viral load occurs in the early phase of illness, close to the time of symptom onset (14, 15). The time to viral clearance is variable across studies, but viral shedding could persist for several weeks after symptom onset, even months in some individuals (16), and may be shorter in young and/or asymptomatic individuals (17). Among hospitalized patients, some observational studies suggest a higher viral load in severe patients compared to nonsevere patients (18, 19), and a recent study found that higher viral load value at hospital admission was independently associated with mortality (20). However, there exist contradictory findings on the impact of disease severity on the duration of virus shedding (15, 21, 22). Besides disease severity, it has been suggested that older age may be associated with a delayed time to viral clearance (23, 24), but this, again, was not found in other studies (15). All together, these elements suggest that viral kinetics could be associated, to some extent, with disease severity. However, the precise assessment of the association between patient’s characteristics, viral kinetics, and survival is unknown and subject to multiple biases emerging from observational studies.

As done for other viral diseases (25, 26), we here used the techniques of mathematical modeling to characterize, in detail, the viral dynamics in the French COVID multicenter prospective cohort of 655 patients admitted to the hospital before April 1, 2020, for which longitudinal data and mortality were collected. We developed a joint model of host/pathogen interaction and survival that captures the heterogeneity of viral patterns observed, reconstructs individual trajectories, and evaluates the association between viral kinetics and death. Finally, we use the model to anticipate the effects of antiviral treatments on viral dynamics and survival.

## Results

### Baseline Characteristics and Virological Data at Admission.

A total of 655 patients were included in this study (Table 1); 478 (77%) were hospitalized in a conventional unit upon admission, and 144 (23%) were admitted in an intensive care unit. The delay between symptom onset and admission ranged from 0 d to 14 d, with a median time of 7 d (interquartile range [IQR]: 3 to 9 d). The majority of patients were male (59%) and under the age of 65 y (59%). Hypertension (39%), obesity (23%), chronic cardiac disease (20%), and diabetes (17%) were the most common comorbidities. Forty percent of patients received oxygen therapy upon admission, with an initial oxygen saturation median value of 95% (IQR: 93 to 97%). Nearly 40% of patients received at least one antiviral treatment during their hospitalization, essentially lopinavir/ritonavir (*n* = 136), hydroxychloroquine (*n* = 62), or remdesivir (*n* = 8); nearly 20% of patients received corticosteroid therapy.

**Table 1.** Demographic, clinical, and biologic characteristics of the 655 patients analyzed

Characteristics	Median (IQR or *n* %)	Missing data (%)
Male gender*	386 (59%)	0 (0%)
Age*	60 (48 to 72)	0 (0%)
Age < 65 y	387 (59%)	0 (0%)
Age ≥ 65 y	268 (41%)	0 (0%)
Time since symptom onset (days)	7 (3 to 9)	6 (1%)
Viral load at admission^†^ (log_10_ copies per mL)	6.3 (4.1 to 8.4)	68 (10%)
Comorbidities		
Hypertension	255 (39%)	9 (1%)
Obesity*	145 (23%)	13 (2%)
Chronic cardiac disease (not hypertension)*	126 (20%)	9 (0%)
Diabetes	111 (17%)	12 (2%)
Chronic pulmonary disease (not asthma)*	72 (11%)	9 (1%)
Asthma	54 (8%)	9 (1%)
Chronic kidney disease	44 (7%)	10 (2%)
Malignant neoplasm	47 (7%)	9 (1%)
Rheumatologic disorder	36 (6%)	12 (2%)
Chronic neurological disorder	30 (5%)	10 (2%)
Chronic hematologic disease	29 (4%)	9 (1%)
Solid organ transplant	16 (3%)	23 (4%)
Chronic liver disease	7 (1%)	9 (1%)
Inflammatory bowel disease	7 (1%)	20 (3%)
Dementia	7 (1%)	10 (2%)
Malnutrition	4 (1%)	12 (2%)
Sickle cell disease	4 (1%)	124 (19%)
AIDS/HIV	0 (0%)	553 (84%)
Splenectomy	3 (0%)	24 (4%)
Clinical characteristics at admission		
Heart rate (beats per minute)	87 (77 to 100)	75 (11)
Respiratory rate (breaths per minute)	21 (18 to 27)	270 (41)
Systolic blood pressure (mmHg)	130 (113 to 145)	74 (11)
Diastolic blood pressure (mmHg)	76 (65 to 85)	74 (11)
Oxygenation on room air	328 (60%)	112 (17)
Oxygen saturation on room air (%)	96 (94 to 98)	0 (0)
Oxygenation on oxygen therapy	215 (40%)	112 (17)
Oxygen saturation on oxygen therapy (%)	95 (93 to 97)	0 (0)
Biological and virological data within the first 2 d after admission		
Haemoglobin (g/dL)		
Platelet count (×10^9^ cells per L)	13.2 (11.8 to 14.4)	209 (32)
WBC count (×10^9^ cells per L)	189 (151 to 244)	209 (32)
Lymphocyte count (×10^9^ cells per L)	5.52 (4.26 to 7.28)	215 (33)
C-reactive protein (mg/L)	0.96 (0.7 to 1.36)	277 (42)
Treatment		
At least one antiviral	240 (39%)	43 (7)
Antibiotic	387 (63%)	40 (6)
Antifungic	32 (5%)	48 (7)
Corticosteroid	108 (18%)	47 (7)

* Baseline risk factors of mortality assessed in our cohort were those identified in the ISARIC international cohort (2), with a prevalence larger than 10% and less than 10% of missing data.

^†^ ±2 d.

**Fig. 1.**
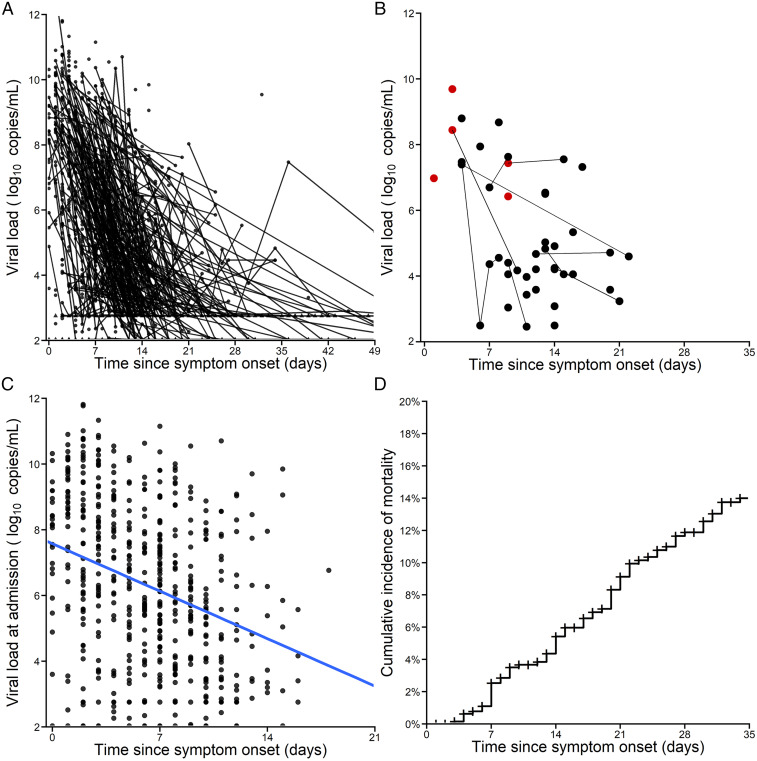
Nasopharyngeal viral load data in 655 patients from the French COVID cohort. (*A*) Longitudinal viral load data expressed in time since symptom onset. (*B*) Viral load data in samples where virus culture was done. Red, positive culture; black, negative culture. The connective lines indicate serial samples from the same patients (44 samples from 37 patients) (*C*) Viral load at admission according to the time since symptom onset; the blue line is the regression line of viral load vs. time. (*D*) Kaplan−Meier curve of cumulative incidence of mortality. Patients lost to follow-up were censored at the last time point where they were known to be alive.

A total of 587 nasopharyngeal swabs were performed within the two first days after admission, and the median viral load value was 6.3 log_10_ copies per mL (IQR: 4.1 to 8.4 log_10_ copies/mL) (Fig. 1*A*). In multivariate regression analysis, viral load at admission was associated with chronic cardiac disease (*P* = 0.009) and a shorter number of days between symptom onset and admission (*P* < 10^−5^), suggesting that patients admitted early after symptom onset had higher viral load than patients arriving later on (Fig. 1*C*) (*SI Appendix*, Table S1).

### Virological Follow-up and Clinical Outcome.

In 284 patients, at least two viral load data were available (i.e., one at hospital admission and at least one during follow-up; *SI Appendix*, Table S2). The median follow-up time was 10 d after admission (ranging from 1 d to 55 d). A total of 78 patients (12%) died during the study follow-up, with a median time to death of 17 d after symptom onset (IQR: 9 to 25 d) (Fig. 1*D*). Overall, only four (5%) deaths occurred later than 35 d after symptom onset; 231 patients were lost to follow-up before that time, essentially due to hospital discharge or transfer to other hospitals, and were then analyzed as censored in survival analyses (see *Materials and Methods*). High levels of viral load (≥6 log_10_ copies per mL) at days 7 and 14 post symptom onset were significantly associated with mortality (*SI Appendix*, Fig. S1).

### Viral Dynamic Modeling.

The best model describing the virological data in terms of Bayesian information criterion (BIC) and residual error incorporated an antigen-dependent stimulation in the elimination of infected cells (*SI Appendix*, Fig. S2 and Tables S3 and S4). In this model, the equation governing the productively infected cells (see *Materials and Methods*) was given by

$$\frac{dI_{2}}{dt}=kI_{1}-\delta I_{2}-\phi \frac{F}{F+\theta }I_{2}.$$ [1]

**Fig. 2.**
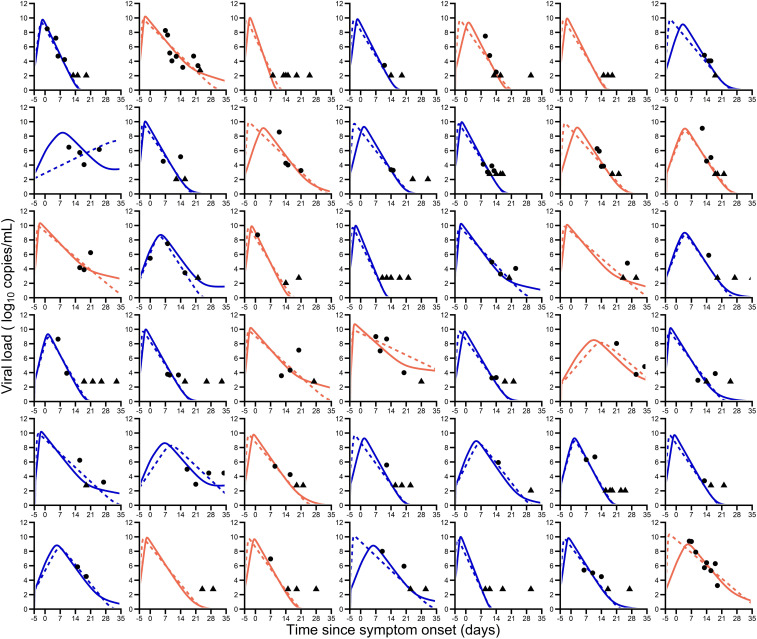
Individual predictions of nasopharyngeal viral kinetics in the 42 patients for which more than three serial samples were available after day 14. The solid and dotted lines (blue, age <65 y; orange, age ≥65 y) are the individual predictions of viral load with the final model and target cell-limited model, respectively (Table 2). The circles are the observed data according to age, and triangles are data below the limit of quantification.

By construction, the minimal and the maximal loss rates of infected cells are given by δ and δ+ϕ, respectively. Following the procedure of covariate selection, only age ≥65 y was associated with a viral kinetic parameter, namely, the maximal decline rate after peak viral load, $\phi \left(P<10^{-4}\right)$. Viral dynamic parameters and their variability were estimated with good precision (Table 2). Our model well recapitulated individual viral kinetics (Fig. 2), and the visual predictive check (VPC) showed that the central trend and the variability predicted by the model were consistent with those observed in the data (*SI Appendix*, Figs. S3 and S4). We also evaluated the result obtained with the target cell-limited model for the sake of comparison (Table 2 and Fig. 2).

**Table 2.** Population parameters of the final joint model of viral dynamics and survival

	Parameter estimates (RSE, %)			
	Final model		Target-cell limited model	
Longitudinal model	Fixed effect	Random effect SD	Fixed effect	Random effect SD
$\beta \left(\text{mL}{\text{.virus}}^{-1}\text{.}\;{\text{d}}^{-1}\right)$	$1.46\times 10^{-5}\left(23.4\right)$	0.65 (26.1)	$4.93\times 10^{-5}\;\left(39.8\right)$	0.95(26.1)
$pT_{0}\left(\text{virus}{\text{.mL}}^{-1}{\text{.d}}^{-1}\right)$	$1.48\times 10^{11}\left(26.8\right)$	$1.82\;\left(7.7\right)$	$8.09\times 10^{10}\;\left(33.5\right)$	$1.74\left(7.7\right)$
*δ* (d^−1^)	$0.33\;\left(30.0\right)$	0.93 (22.3)	−	−
$\delta_{\mathrm{age}}\left(<65\right)\left(d^{-1}\right)$	−	−	$1.09\;\left(7.8\right)$	$0.39\left(8.7\right)$
$\delta_{\mathrm{age}}\left(\geq 65\right)\left(d^{-1}\right)$	−	−	0.84 (22.8)	−
$\phi_{\mathrm{age}}\left(<65\right)\left(d^{-1}\right)$	$0.92\;\left(8.7\right)$	0.20 (31.2)	−	−
$\phi_{\mathrm{age}}\left(\geq 65\right)\left(d^{-1}\right)$	0.65 (23.3)	−	−	−
$\theta \left(F.mL^{-1}\right)$	70 (80.8)	−	−	−
$T_{\inf}\left(d\right)$	$4.8\;\left(3.2\right)$	0.12 (14.7)	$5.4\;\left(9.0\right)$	0.16(14.7)
$\sigma \left(\text{virus}{\text{.mL}}^{-1}\right)$	1.49 (4.0)	−	1.58 (4.7)	−
				
Male gender	2.55 (25.2)	-	2.54 (28.5)	-
Age ≥65 y	2.58 (37.9)	-	2.59 (24.6)	-
Chronic pulmonary disease	2.31 (36.8)	-	2.27 (38.7)	-
Viral load (log_10_ virus.mL^−1^)	1.31 (17.0)	-	1.30 (17.1)	-

*β*, infection rate; *δ*, loss rate of infected cells; *p*, rate of viral production; *ϕ*, maximal rate of immune cell clearance; *θ*, F concentration giving 50% of *ϕ*; *T*_*inf*_, time to infection; *σ*, residual variability; RSE, relative SE.

**Fig. 3.**
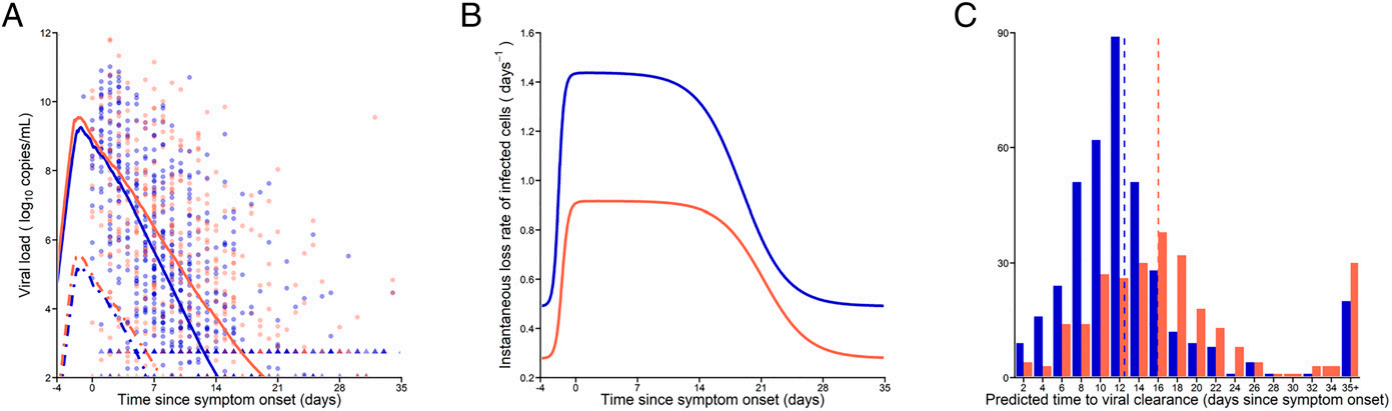
Description of the individual viral load kinetic profiles. (*A*) Median of the individual predicted viral load kinetics. Solid lines, total viral load levels; dashed lines, infectious virus. Dots represents the observed data, and triangles are data below the limit of quantification. (*B*) Median of the predicted instantaneous loss rate of productively infected cells. (*C*) Distribution of the predicted time to viral clearance in the patients. Dashed lines represent the predicted median of time to viral clearance. Viral kinetic parameters were obtained by using the EBE (see *Materials and Methods*). Blue, patients aged <65 y; orange, patients aged ≥65 y.

Viral dynamic parameters predicted that viral load peaked, on average, 1.1 d before symptom onset, with values of ∼9.8 log_10_ copies per mL, with no difference between patients aged ≥65 y and those aged <65 y. In this model, the effective loss rate of productively infected cells was further enhanced by model-predicted differences in the action of immune effector *F*. Maximal effect of this immune response, ϕ, was estimated to be 0.92 d^−1^ for patients aged <65 y and 0.65 d^−1^ for patients aged ≥65 y. Therefore, the loss rate of infected cells varied from δ = 0.33 d^−1^ at the beginning of the infection to $\mathrm{\delta +}\phi =1.25\;d^{-1}$ and 0.98 d^−1^ in patients <65 y and ≥65 y, respectively, at the peak of the infection, when the effect of the immune response was maximal, as illustrated in Fig. 3 *A* and *B*. This allowed us to capture a biphasic decline of the virus after peak viral load, where a rapid age-dependent decline was followed by a slower decline rate due to lower antigen stimulation. This corresponded to a half-life of infected cells decreasing from 50 h (IQR: 30 to 52 h) in all patients to 13 h (age <65 y; IQR: 11 to 14 h) and 17 h (age ≥65 y; IQR: 14 to 17 h) (Fig. 3*B*). The predictions obtained by the target cell-limited model gave close estimates, with a mean half-life of infected cells of 15 and 20 h in patients aged <65 y and those aged ≥65 y, respectively (Table 2). As a consequence, the predicted time to viral clearance occurred earlier in patients aged <65 y, with a median time of 13 d after symptom onset (IQR: 10 to 15 d) as compared to 16 d (IQR: 12 to 20 d) in patients aged ≥65 y (*P* < 10^−4^) (Fig. 3*C*).

In the samples where virus was cultured, extended viral shedding was not associated with culture positivity, with all cultures of viral load assessed more than 10 d after symptom onset being negative (Fig. 1*B*). As an exploratory analysis, we evaluated the association between antiviral or corticosteroid treatment and viral load decay. No significant association was found between antiviral treatment and time to viral clearance. Corticosteroid had no effects on the time to viral clearance in aged patients; however, younger patients treated with corticosteroids had a longer time to viral clearance as compared to young untreated patients (*P* = 0.01; *SI Appendix*, Fig. S5; see *Discussion*).

### Alternative Models and Sensitivity Analyses.

Alternative models of the immune response did not improve the data fitting, and parameter estimates were close to those obtained with the final model (*SI Appendix*, Tables S5 and S6). In the subset of 76 individuals where antibody could be measured, the median time to seroconversion was 12 d (*SI Appendix*, Fig. S6). We therefore also tested models assuming an increase in the loss rate of productively infected cells after 12 d, but these models did not lead to any improvement of the fitting criterion (*SI Appendix*, Table S6).

To evaluate reliability of our final model predictions, we also performed sensitivity analyses, varying fixed parameters k between 1 and 5 d^−1^ and c between 5 and 20 d^−1^. The parameter estimates and the BIC were stable in all tested values, except when k was lower than 3 d^−1^ (*SI Appendix*, Fig. S7).

### Association between Viral Dynamics and Mortality.

**Fig. 4.**
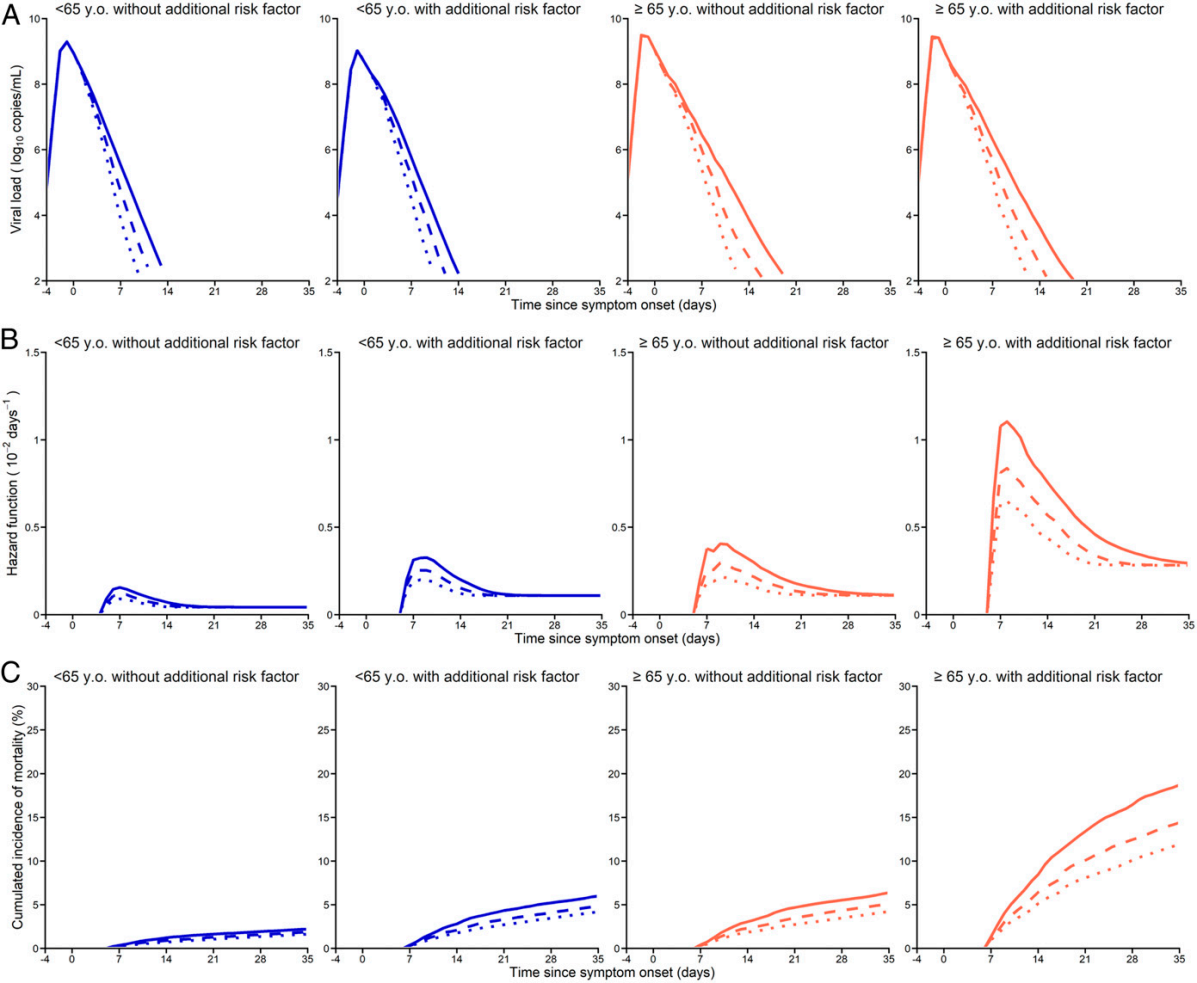
Individual viral load and survival profiles and predictions according to the initiation of a putative antiviral treatment initiated at admission. (*A*) Median of the individual predicted viral load, *V*(*t*) (Eqs. **1**–**3**); y.o., years old. (*B*) Median of the predicted instantaneous hazard function, *h*(*t*). (*C*) Median of the predicted death rate, 1 − *S*(*t*) (Eq. **4**). Solid lines, predicted profile without treatment; dashed lines, treatment with 90% efficacy; dotted lines, treatment with 99% efficacy; blue, patients aged <65 y; orange, patients aged ≥65 y. The profiles are calculated in each of the following population of patients: age <65 y and absence of other significant risk factor (male gender or chronic pulmonary disease, *Left*), age <65 y and presence of at least one risk factor (*Center Left*), age ≥65 y and absence of other risk factor (*Center Right*), and age ≥65 y and presence of at least one other significant risk factor (*Right*).

In the 74 individuals who died within 35 d from symptom onset, the model predicted that the viral load was below the limit of quantification/detection in 23% (17/74) of the cases (*SI Appendix*, Fig. S8). In 39% of individuals (29/74), the viral load predicted by the model was higher than 6 log_10_ copies per mL at time of death. In multivariate analyses, the risk factors associated with survival were chronic pulmonary disease, age ≥65 y, and male gender (*SI Appendix*, Table S7). Using a joint model adjusting for these risk factors, the viral load was significantly associated with survival (hazard ratio [HR] = 1.31, *P* < 0.001), showing that viral load dynamics was an independent predictor of death. In the final joint model, the hazard ratios associated with age ≥65 y, male gender, and chronic pulmonary diseases were equal to 2.58, 2.55, and 2.31, respectively, showing that the presence of any of these risk factors were associated with a large increase in the risk of death (all *P* < 0.01). The joint model could well recapitulate the impact of viral load and risk factors on survival rate, with patients characterized by older age and a prolonged viral shedding showing a rapid decline of their predicted survival rate, in particular when there was another risk factor (i.e., male gender or chronic pulmonary disease; Fig. 4 and *SI Appendix*, Fig. S9).

Here, as well, we tested alternative models to test the robustness of our predictions. Assuming that the hazard function, *h*(*t*), only increased after a fixed time of 7 d or 10 d after infection worsened the fitting criterion, but predicted a similar association between viral load dynamics and survival. Similar results were obtained with alternative models where *h*(*t*) could increase upon infection or symptom onset, consistent with the interpretation that late, but not early, viral load may be associated with outcome (*SI Appendix*, Table S8).

### Antiviral Treatment Simulation.

To get a better sense of the specific impact of viral load, we further stratified our population according to age and presence of at least one risk factor. Then we evaluated the model predictions with a putative potent antiviral therapy capable of reducing viral production,p, by 90% or 99%, that would be initiated upon hospital admission. A treatment inhibiting 90% of the viral production could shorten the median time to viral clearance by 2 d (IQR: 1.5 to 2.2 d) in patients aged <65 y and by 2.9 d (IQR: 1.9 to 3.8 d) in patients aged ≥65 y (Fig. 4*A*). Considering a treatment blocking 99% of viral production, the effects would be further improved in patients aged ≥65 y, with a median reduction in time to viral clearance of 5.4 d (IQR: 3.7 to 6.9 d).

To calculate the impact of reduced viral load levels on mortality, we assumed that viral dynamics was on the causal pathway for mortality, and thus the association between viral load and mortality was fixed to the value found in our original cohort of patients that did not receive any effective antiviral therapy. With a treatment blocking 90% of viral production, the accelerated viral decline would translate, in this model, into mortality rates reduced from 2.2 to 1.9% in patients below the age of 65 y with no additional risk factor, and from 6.0 to 4.8% in those with at least one risk factor (i.e., either being male or having a chronic pulmonary disease). The effects of treatment would be more sensible in patients above the age of 65 y, with a mortality reduced from 6.4 to 5.0% for those without additional risk factor and from 19 to 14% in those having at least one other risk factor. With a treatment efficacy of 99%, the effects would be even larger, with a decrease in mortality from 19 to 12% in patients above the age of 65 y having one additional risk factor (Fig. 4*C*).

## Discussion

To the best of our knowledge, this is the largest analysis of prospective nasopharyngeal SARS-CoV-2 viral dynamic data in hospitalized patients. A mathematical model of viral dynamics accounting for age- and time-dependent effects in the loss rate of infected cells could well reproduce the variability of the patterns observed, and found that older patients had a slower decline of virus after peak viral load. Using joint modeling, a statistical approach to assess the effect of a time-dependent covariate on the hazard function, we could show that the viral load was associated with death, even after adjustment of risk factors such as chronic pulmonary disease, older age, and male gender. This result was robust to various changes in model assumptions, including the parameterization of the second-phase decline. This shows that viral load kinetics could, in addition to other established risk factors, help identify most-at-risk patients during hospitalization. Accordingly, it suggests that approaches reducing viral load levels could reduce mortality, and our model can be used to quantify the magnitude of such an effect.

Our population was in line with previous series of hospitalized patients, with a population predominantly male (59%), aged 65 y and more (41%), with comorbidities that were mostly hypertension (39%), obesity (23%), and diabetes (17%) (2, 6, 7). The first striking observation in our series, as previously suggested (20), was the strong correlation between the viral load at admission and the time since symptom onset, suggesting that the peak viral load was close to the time of symptom onset. To explore this possibility in greater detail, we used a mathematical model of viral kinetics. The model providing the best fit to the data was a target cell-limited model in which the loss rate of infected cells was antigen dependent. Although only a few viral load data were available in the first 7 d after symptom onset, and the exact time of peak viral load could not be precisely observed, our modeling predictions suggested that the mean peak viral load was close to symptom onset (15, 27). It is noteworthy that only limited information was available in the first days from symptom onset (*SI Appendix*, Table S2), which means that these estimates represent a typical trajectory in our population, and may hide variability in the early kinetics that could not be observed in our data. Using the target cell-limited model, results also nonetheless indicated a high within-host reproduction, with a mean *R*_0_ of 36. Results obtained in previous reports suggested lower levels of *R*_0_, in a range 5 to 15 (28–30). In nonhuman primates, which do not show severe infection, we estimated *R*_0_ in the nasopharynx to be about 5.6 (95% CI: 1.3 to 21) (31). Whether the high value found here is a consequence of the disease severity of our population as compared to other reports or is artifactual due to the limited information in the very early phase of the disease will require more investigation. Whatever the exact value of *R*_0_, the fact that the viral load observed in early admitted patients was very high and could be above 10 log_10_ copies/mL is consistent with an intense replication rate of the virus that coincides with symptom onset.

The viral load after peak declined in a biphasic manner, consistent with observations made by He et al. (15). The first phase of viral decline was rapid and age dependent, with a rate equal to 1.25 and 0.98 d^−1^ in patients aged <65 y and ≥65 y, respectively, when the effect of the immune response was maximal (Fig. 3*B*). This corresponded to half-life of infected cells decreasing from 50 h to 13 h (age <65 y) and 17 h (age ≥65 y). After this first decline, a second phase of viral decline ensued that was slower, with rate *δ*. As we relied only on viral data and did not have access to immunological data, we could not assess which component of the immune response was most likely involved in the time-dependent clearance rate of infected cells. In the subset of individuals in which antibody could be measured, the median time to seroconversion was 12 d after symptom onset (*SI Appendix*, Fig. S6). However, models that assumed an increase in the loss rate of infected cells after day 12 did not improve data fitting (*SI Appendix*, Table S6). The reduced loss rate of infected cells could stem from a lower antigen stimulation, as proposed in our model, or from other immunological features, such as immune exhaustion, and suggests that seroconversion does not lead to a dramatic acceleration of viral clearance in these patients. Here we did not identify complex patterns of viral dynamics such as described in refs. 32–34; however, this may also be due to the fact that such models require intensive sampling measurements, which can hardly be done outside the context of small specific clinical or preclinical studies. Antiviral treatment and corticosteroids could, in theory, play a role in this viral decay, but could not be properly estimated in this observational study. However, we now know that hydroxychloroquine and lopinavir/ritonavir are highly unlikely to have any antiviral activity in SARS-CoV-2−infected patients (35–37). Regarding corticosteroids, a small effect toward delayed time to viral clearance in young treated as compared to young untreated patients was found.

As a consequence of their faster viral decline rate, the predicted time to viral clearance occurred earlier in patients aged <65 y, with a median time of 13 d postsymptom onset as compared to 16 d in patients aged ≥65 y (*P* < 10^−4^). These median values nonetheless mask important heterogeneity across patients. For instance, 25% of patients aged <65 y had detectable viral loads 15 d after symptom onset, while this increased to 20 d in patients aged ≥65 y. These results show the importance of younger age, probably reflective of a more effective immune response, and are in line with previous reports (19, 38). It is important to note that a longer period of viral excretion may not necessarily translate into a similarly long infectiousness period. In our data, all viral cultures from samples collected more than 10 d after symptom onset or associated with viral load lower than 6 log_10_ copies per mL were negative (Fig. 1*B*). Even if culture results are not a direct measure of the infectiousness, they nonetheless suggest that the period during which an individual is contagious is probably short, even if virus continues to be excreted (16, 39, 40). In fact, our model predicted that the median time to achieve 6 log_10_ copies/mL was 6.5 and 8.3 d in patients aged <65 y and ≥65 y, respectively (*P* < 10^−4^).

Next, we aimed to assess the factors associated with death. In multivariate analyses, age ≥65 y, male gender, and chronic pulmonary diseases were strongly associated with survival (all HR > 2). By using a joint model, a method used to capture the effects of time-dependent covariate on survival, viral kinetics was also identified as an independent predictor of death (HR = 1.31, *P* < 10^−3^). Although the effects of viral load were probably not as a dramatic as those of risk factors, and its causality has not been yet established, our results nonetheless suggest that reducing viral dynamics could have a sensible effect on mortality. To study the effects of reducing viral load in more detail, we conducted simulations assuming that an antiviral treatment reducing 90% or 99% of viral production could be readily initiated upon hospital admission. The largest effects were obtained in patients aged ≥65 y and having at least one other risk factor, with a reduction of the time to viral clearance of 2.9 and 5.4 d, respectively. Assuming that the effects of viral dynamics on survival would remain similar to what was obtained here in patients receiving no effective antiviral treatments, this would translate to predicted mortality rates at day 35 of 14% and 12%, respectively, as compared to 19% in our cohort. In other populations of younger patients or without additional risk factors, the effects were predicted to be much less dramatic. Interestingly, we here considered, for the sake of simplicity, treatments blocking viral production, but largely similar results could be obtained with drugs that would block infection, as is the case for monoclonal antibodies. This level of 90% is not out of reach, and roughly corresponds to drug concentrations being 10 times higher than their EC_50_ (concentration for which 50% of maximum effect is obtained), which are standard for antiviral drugs in other infections [e.g., HIV, hepatitis B virus , hepatitis C virus, Ebola (41)]. Given the fact that peak viral load occurs early, as discussed above, it is likely that aggressive strategies that could identify patients earlier than in our study (where admission occurred, on average, 7 d after symptom onset) could have even better results. Our prediction also implicitly hypothesizes that viral load is in the causal pathway of mortality and that effective antiviral treatment, by reducing viral load, would reduce mortality rate. This assumption has been verified for other acute severe viral infections for which effective antiviral strategies exist, such as Ebola virus (8, 42). In the case of SARS-CoV-2, several antiviral drugs have been tested, but the effects on viral kinetics or even symptom evolution remain modest, even though promising results were recently found with monoclonal antibodies (43). It is also possible that viral replication in the lower respiratory tract (LRT) is a better predictor of outcome than nasopharyngeal viral load (44). Here our data showed a correlation between viral load values in both compartments (*SI Appendix*, Fig. S10). However, the predictive value of LRT could not be tested, due to the fact that patients having LRT were not representative and had a much larger mortality rate than the general population (*SI Appendix*, Fig. S10).

To conclude, our analysis reveals that hospitalized patients are characterized by a high viral load peak close to symptom onset in a majority of patients, followed by a biphasic decline, with age being significantly associated with a delayed viral shedding. Using a joint model, we showed that viral load was an independent factor associated with death. Our results demonstrate the importance of viral dynamics to identify at-risk patients and suggest that strategies aiming to accelerate viral clearance could decrease mortality in these patients.

## Materials and Methods

### Ethics.

Hospitalized patients with a laboratory-confirmed SARS-CoV-2 infection were enrolled in the French COVID cohort (registered in clinicaltrials.gov NCT04262921) (45). The study was conducted with the understanding and the consent of each participant or its surrogate. The study was sponsored by French National Institute of Health and Medical Research, and was supported by the Research & Action emerging infectious diseases (REACTing) consortium (46) and the French Ministry of Health. Ethics approval was given on February 5 by the French Ethics Committee CPP-Ile-de-France VI (ID RCB: 2020-A00256-33).

### Patients and Data Collection.

We analyzed the results obtained from patients enrolled in the cohort between February 5 and April 1, 2020, hospitalized in 18 different hospitals and for whom nasopharyngeal swabs were available. Data were collected from the French modified version of the open-access Case Report Form of the Clinical Characterization Protocol for Severe Emerging Infections of International Severe Acute Respiratory and Emerging Infection Consortium (ISARIC) (47). Data at admission time included demographic characteristics (age and gender), time since symptom onset, presence of various comorbidities, and clinical and biological markers. Survival data after hospital admission were collected up to 60 d postadmission. Patients lost to follow-up were censored at the last date of observation.

### Viral Load Assessment.

Nasopharyngeal swabs were collected at admission and during patient’s follow-up. In a subset of patients, viral load in the LRT was also available (*SI Appendix*, Table S2). Viral load was determined by real-time semiquantitative RT-PCR, using either the Charité World Health Organization (WHO) protocol (testing E gene and RdRp) or the Pasteur institute assay (testing E gene and two other RdRp targets: IP2 and IP4) (48, 49). Results were provided in cycle threshold (Ct) and transformed into log_10_ RNA copies per mL using the relationship assessed by Pasteur Institute for both E and IP4 genes (48) IP4 viral load value was used as a reference (Pasteur institute assay), or E gene (Charité WHO protocol). For E gene, a corrective factor was applied to correct for the differences observed between the viral load from the two genes (*SI Appendix*). Ct values above 37 (corresponding to a viral load of ∼2 log_10_ for E gene and 2.7 log_10_ for IP4) were considered as below the limit of detection.

### Infectious Virus.

For a subset of patients, viral isolation was performed in a biosafety level 3 (BSL-3) laboratory. Briefly, for each sample, 200 µL of virus transport media containing the nasopharyngeal swab was diluted into 800 µL of Dulbecco’s modified Eagle’s medium (DMEM, Gibco), filtered through a 0.45-µm filter (Sartorius); then 500 µL of the filtered material was inoculated to 50,000 Vero E6 cells (reference CRL-1586, ATCC) plated in a 24-well plate. After 1 h at 37 °C, 500 µL of DMEM containing 4% of FBS (fetal bovine serum, Gibco) was added to each well. At day 6 postinfection, wells were screened for cytopathogenic effect (CPE), and, for all wells with signs of CPE, an RT-PCR targeting both E and S genes of SARS-CoV-2 (RealStar SARS-CoV-2 RT-PCR, Altona) was performed on 50 µL of supernatant to confirm the success of viral isolation.

### Risk Factors Associated with Viral Load and Survival.

We explored the effects of risk factors associated with higher mortality in hospital previously found by ISARIC, a consortium of large cohort studies of hospitalized patients (2), namely, age (≥65 y), male gender, chronic cardiac disease, chronic pulmonary disease, chronic kidney disease, obesity, dementia, moderate/severe liver disease, chronic neurological disease, or malignancy. Among them, only those with less than 10% of missing data and more than 10% of prevalence in our dataset were explored in our analyses. Thus, only age ≥65 y, male gender, chronic cardiac disease, chronic pulmonary disease, and obesity were evaluated in our analyses (Table 1).

### Factors Associated with the Viral Load at Admission.

We aimed to evaluate the factors associated with the viral load at admission (defined as the first viral load available within 2 d of admission) in a linear regression model, according to the risk factors described above and the time since symptom onset. All factors associated with *P* ≤ 0.1 in univariate analyses were tested in multivariate analyses. Backward elimination was used, and *P* < 0.05 was considered statistically significant. In multivariate analyses, missing data for continuous comorbidities were imputed to the median population value. For categorical risk factors, the values were imputed randomly with a probability equal to the prevalence of each category in the population.

### Viral Dynamic Model.

#### Model equations.

We used a target cell-limited model with an eclipse phase (8, 25, 26) . The model includes three types of cell populations: target cells (T), infected cells in an eclipse phase $\left(I_{1}\right)$, and productively infected cells $\left(I_{2}\right)$. The model assumes that target cells are infected at a constant infection rate β (milliliters per virion per day). Once infected, cells enter an eclipse phase and become productively infected after a mean time 1/k (d). We assume that productively infected cells have a constant loss rate, noted $\delta \left(d^{-1}\right)$. Infected cells produce p viral particles per day (virus per day), but only a fraction of them, μ, is infectious, and the virus particles can either be infectious, noted $V_{i}$, or noninfectious, noted $V_{ni}$. We assumed that viral load, as measured by RNA copies, is the sum of infectious and noninfectious viral particles, both cleared at the same rate, c. The model can be written as

$$\frac{dT}{dt}=-\beta V_{i}T$$

$$\frac{dI_{1}}{dt}=\beta V_{i}T-kI_{1}$$

$$\frac{dI_{2}}{dt}=kI_{1}-\delta I_{2}$$ [2]

$$\frac{dV_{i}}{dt}=p\mu I_{2}-cV_{i}$$

$$\frac{dV_{ni}}{dt}=p\left(1-\mu \right)I_{2}-cV_{ni}.$$

The basic reproductive number *R*_0_, defined by the number of secondary infected cells resulting from one infected cell in a population of fully susceptible cells, *T*_0_, is defined by $R_{0}=\beta pT_{0}\mu /c\delta$.

In order to evaluate the role of an immune response, we added to the model a dimensionless compartment *F* representing an immune response stimulated by viral antigens (50), defined as follows:

$$\frac{dF}{dt}=I_{2}-d_{f}F.$$ [3]

We considered several scenarios where F either acts by reducing viral infectivity and leads to cells refractory to infection (51–53), decreases the rate of viral production (54, 55), decreases the infection rate (25), or increases the viral clearance or the loss rate of infected cells (55–57) (*SI Appendix*, Fig. S2). Following the class of models that we evaluated in previous works (30), we used similar parameterizations for the different models (*SI Appendix*), with a nonlinear and saturable effect for *F* defined by ϕ(F/F+θ). Thus, in this model, ϕ represents the maximal potential effect of the immune response, and θ is the level of F required to achieve 50% of this maximal effect.

#### Assumptions on parameter values.

To ensure parameter identifiability, a number of parameters had to be fixed. Virion clearance rate, c, was fixed to 10 d^−1^ similar to what has been performed in ref. 30. We assumed that the loss rate of immune effectors was slower than viral clearance, and we fixed *d*_*f*_ = 0.4 d^−1^ (8). As only the product $p\times T_{0}$ is identifiable, we fixed the density of susceptible epithelial cells, *T*_0_, to $1.33\times 10^{5}$ cells per mL. This assumption stems from the fact that there are $∼4\times 10^{8}$ epithelial cells in the upper respiratory tract (URT), that the URT has a volume of 30 mL, and that 1% of cells express ACE2 and TMPSS receptor (58), which are used by the virus to bind target cells. The proportion μ of infectious viruses was fixed to 10^−4^ of the total RNA viral load, which was the upper bound found in animal models (31).

#### Initial conditions at infection.

In each patient, we estimated the time of infection, noted *t*_inf_. To ensure identifiability, we assumed that, at *t* = *t*_inf_, there was exactly one productively infected cell in the entire URT. Thus, for *t* ≤ *t*_inf_, *T* = *T*_0_; *I*_1_ = 0; *I*_2_ = 1/30; and *V* = 0. We assumed a maximal duration of 14 d between the infection and the onset of symptoms, and thus *t*_inf_ was bounded between 0 and 14 d before symptom onset (14).

#### Parameter estimation and fitting assessment.

The structural model used to describe the observed log_10_ viral loads $Y_{ij}$ of the *i*th subject at time *t*_*ij*_ is

$$Y_{ij}=log_{10}V\left(t_{ij},\;\psi_{i}\right)+e_{ij},$$

where $V\left(t_{ij},\;\psi_{i}\right)$ is the viral load predicted by the model at time *t*_*ij*_, $\psi_{i}$ is the vector of parameters of subject *i*, and $e_{ij}$ is an additive residual Gaussian error term of constant SD, $\sigma_{ij}$. Individual parameters $\psi_{i}$ follow a log-normal distribution,

$$\psi_{i}=\gamma \times exp\left(\eta_{i}\right),$$

where *γ* indicates the fixed effects and $\eta_{i}$ are the random effects following a normal centered distribution with a diagonal variance−covariance matrix Ω.

Parameters were estimated by computing the maximum likelihood estimator using the stochastic approximation expectation–maximization (SAEM) algorithm implemented in Monolix Software 2018R2 (http://www.lixoft.eu/). Goodness-of-fit was assessed by visual inspection of VPC (see more details in *SI Appendix*, *Supplementary Information Text*) (59).

#### Model building strategy for viral dynamics.

We used only viral load data to construct the viral dynamic model following the strategy below:

1) The target cell-limited model (Eq. **2**) was used to estimate the parameters.
2) The five models with immune effects (models B through F; *SI Appendix* and Eqs. **2** and **3**) were tested. The model providing both the lowest BIC and the lowest residual errors was retained, provided that the SEs were not worsened and the goodness-of-fit graphics were adequate.
3) The impact of risk factors was then assessed (see above). Covariates were screened using empirical Bayes estimates (EBE) of individual parameters using nonparametric tests, and those with *P* < 0.1 were included in the model. Backward elimination was used, and *P* < 0.05 was considered statistically significant.
4) Random effects with an SD < 0.1 or associated with a relative SE of greater than 100% were deleted by using a backward procedure and were kept out if the resulting BIC did not increase by more than two points.

### Joint Model to Evaluate the Impact of Viral Dynamics on Survival.

#### Baseline risk factors associated with survival.

Let $X_{i}$ and $C_{i}$ denote survival and censoring times for patient i, respectively, and $T_{i}$ denote the last time of observation: $T_{i}=\min\left(X_{i},C_{i}\right)$, $\delta_{i}=1_{\left\{X_{i}\leq C_{i}\right\}}$. We used a parametric exponential survival model to characterize the baseline hazard function, with constant rate λ. Consistent with what was done above, the risk factors associated with a *P* ≤ 0.1 in univariate analyses were tested in multivariate analyses. Backward elimination was used, and variables were removed one by one until no improvement of BIC was obtained.

#### Assessing the impact of viral load on survival.

Finally, we aimed to assess the impact of viral dynamics on survival using a joint model, where the parameters of both viral load dynamic and survival are analyzed simultaneously to limit the bias due to dropout (8, 59, 60). In this model, the hazard function for patient *i* at time $t,\;h_{i}\left(t|\psi_{i}\right)$is the instantaneous hazard function in patient *i* at time *t*,

$$h_{i}\left(t|\psi_{i}\right)=\lambda \times \text{exp}\left(\sum_{j=1}^{r}\gamma_{j}\times z_{i}^{j}+\nu \times log_{10}V\left(t,\psi_{i}\right)\right),$$

where *r* are the significant risk factors found in the survival analysis above, $z_{i}^{j}$ denotes the presence or the absence of the risk factor *j* in patient *i*, and $\text{exp}\left(\gamma_{j}\right)$ denotes the hazard ratio associated with the risk factor *j.* Finally, ν denotes the impact of the current (log) viral load value in patient on the hazard function, with ν=0 implying that viral load has no impact on the hazard function and ν>0 implying that the current viral load value increases the hazard function. Using the same notation as before, exp(ν) is the hazard ratio of the current viral load value.

For the sake of identifiability, we assumed that $h_{i}\left(t|\psi_{i}\right)$ was equal to 0 up until time of admission, and the survival rate up to time *t* in patient *i*, noted $S_{i}\left(t|\psi_{i}\right)$, was then obtained using the following formula:

$$S_{i}\left(t|\psi_{i}\right)=\exp\left[-\int_{0}^{t}h_{i}\left(u|\psi_{i}\right)du\right].$$ [4]

The joint likelihood of the longitudinal and the survival data is then maximized using the SAEM algorithm following the methodology developed in refs. 8, 59, 60.

#### Predicting the impact of an effective antiviral treatment on viral kinetics and survival.

The joint model was also used to simulate the effect of an antiviral treatment on viral kinetics and survival. We simulated this impact on the patients from the cohort, using their individual parameters previously estimated. We considered the effect of a treatment that would be initiated at the time of admission, with an antiviral efficacy, noted ε, in blocking viral production, *p*, equal to 90% or 99%. For each scenario, we plotted the median viral load and the median survival value, and the results were stratified according to the presence or absence of significant risk factors.

#### Sensitivity analyses and robustness.

Because some parameters were not estimated, we also reestimated parameter values of the final model assuming different values of *k* and *c* ranging from 1 d^−1^ to 5 d^−1^, and from 5 d^−1^ to 20 d^−1^, respectively. We also tested a number of other parameterizations of the loss rate of infected cells. In particular, as the median date of antibody apparition was equal to 12 d after symptom onset in the subset of individuals where antibody had been measured (*SI Appendix*, Fig. S6), we tested models where the loss rate of infected cells could increase after day 12 (*SI Appendix*, Table S5).

We also considered different assumptions for the change in the instantaneous hazard rate for the survival component of the model, with models assuming that the hazard function could be different from 0 as early as the predicted time of infection, the onset of symptom, or after a fixed period of 7 d or 10 d after symptom onset (*SI Appendix*, Table S8).

To facilitate model comparisons, we also gave the results of the target cell-limited model assuming no change in the loss rate of infected cells over time and an effect of age on the loss rate of infected cells.

## Supplementary Material

Supplementary File

Supplementary File

Supplementary File

Supplementary File

## Data Availability

Anonymized spreadsheet of data has been deposited in Figshare (https://doi.org/10.6084/m9.figshare.13365398.v1).
