# Supplementary material for: Modeling SARS-CoV-2 viral kinetics and association with mortality in hospitalized patients from the French COVID cohort
Source: Proc Natl Acad Sci U S A. 2021 Feb 3;118(8):e2017962118. doi: 10.1073/pnas.2017962118 (PMC7929555; doi:10.1073/pnas.2017962118)
Supplement: Supplementary File [file pnas.2017962118.sapp.pdf]

Co-author 1			
Name	***Last name***	***Mail adress @***	***Affiliation***
Raphael	BORIE	raphael.borie@aphp.fr	Paris - Bichat - SMIT
Etienne	DE MONTMOLLIN	etienne.demontmollin@aphp.fr	Paris - Bichat - Réanimation
Duc	NGUYEN	duc.nguyen@chu-bordeaux.fr	Bordeaux - SMIT
Nicolas	BENECH	nicolas.benech@chu-lyon.fr	Lyon - SMIT
Elisabeth	BOTELHO-NEVERS	elisabeth.botelho-nevers@chu-st-etienne.fr	Saint Etienne - SMIT
Olivier	EPAULARD	OEpaulard@chu-grenoble.fr	Grenoble - SMIT
Camille	CHASSIN	cchassin@ghnd.fr	Bourgoin-Jallieu - Médecine interne
Aldric	MANUEL	amanuel@ch-annecygenevois.fr	Annecy - SMIT
François Xavier	CATHERINE	francois-xavier.catherine@chu-dijon.fr	Dijon - SMIT
Manuel	ETIENNE	Manuel.Etienne@chu-rouen.fr	Rouen - SMIT
Julien	POISSY	julien.poissy@chru-lille.fr	Lille - Réanimation
Eric	SENNEVILLE	esenneville@ch-tourcoing.fr	Tourcoing - SMIT
Karine	FAURE	karine.faure@chru-lille.fr	Lille - SMIT
Clotilde	ALLAVENA	Clotilde.ALLAVENA@chu-nantes.fr	Nantes - SMIT
Stéphane	SALLABERRY	ssallaberry@ch-annecygenevois.fr	Annecy - Réanimation
Elisa	DEMONCHY	demonchy.e@chu-nice.fr	Nice - SMIT
Fabrice	LAINE	Fabrice.Laine@chu-rennes.fr	Rennes - SMIT
Valentine	CAMPANA	Valentine.CAMPANA@chu-martinique.fr	Fort de France - SMIT
Julie	CHAS	julie.chas@aphp.fr	Paris - Tenon -SMIT

Antoine	KIMMOUN	a.kimmoun@chru-nancy.fr	Nancy - Réanimation
François	GOEHRINGER	f.goehringer@chru-nancy.fr	Nancy - SMIT
Erwan	L'HER	erwan.lher@chu-brest.fr	Brest - Réanimation
Vincent	LE MOING	v-le_moing@chu-montpellier.fr	Montpellier - SMIT
Alexa	DEBARD	debard.a@chu-toulouse.fr	Toulouse - SMIT
Paul	LOUBET	Paul.LOUBET@chu-nimes.fr	Nimes - SMIT
Hugues	CORDEL	hugues.cordel@aphp.fr	Bobigny - Avicenne - SMIT
Laurent	BITKER	laurent.bitker@chu-lyon.fr	Lyon - Réanimation
Felix	DJOSSOU	felix.djossou@ch-cayenne.fr	Cayenne - SMIT/Réanimation
Vincent	DINOT	v.dinot@chr-metz-thionville.fr	Metz - Réanimation
Rafael	MAHIEU	Rafael.Mahieu@chu-angers.fr	Angers - SMIT
Charline	VAUCHY	cvauchy@chu-besancon.fr	Besancon - SMIT
Martin	MARTINOT	Martin.martinot@ch-colmar.fr	Colmar - SMIT
Gwenhaël	COLIN	gwenhael.colin@chd-vendee.fr	La Roche Sur Yon - Infectiologie
Denis	GAROT	d.garot@chu-tours.fr	Tours - Réanimation
Cécile	GOUJARD	cecile.goujard@aphp.fr	Kremlin-Bicêtre -SMIT/Médecine interne
Isabelle	ENDERLE	Isabelle.ENDERLE@chu-rennes.fr	Rennes - Gynécologie
Séverine	ANSART	severine.ansart@chu-brest.fr	Brest - SMIT
Guillermo	GIORDANO	GIORDANO.Guillermo@ch-avignon.fr	Avignon - SMIT
Vincent	PEIGNE	vincent.peigne@ch-metropole-savoie.fr	Chambery - SMIT
Sylvain	DIAMANTIS	Sylvain.diamantis@ghsif.fr	Melun - SMIT
Elodie	CURLIER	elodie.curlier@chu-guadeloupe.fr	Guyane - Guadeloupe -Réanimation - SMIT
Jean-	LELIEVRE	jean-daniel.lelievre@aphp.fr	Créteil - Mondor - SMIT

Daniel			
Nadia	SAIDANI	n.saidani@ch-cornouaille.fr	Quimper - MIIS
Victoria	MANDA	victoria.manda@aphp.fr	Paris - Lariboisière - SMIT
Adrien	LEMAIGNEN	adrien.lemaignen@chu-tours.fr	Tours - SMIT
Cecile	AZOULAY	cecile.azoulay@aphp.fr	Paris - Cochin - CIC Vaccinologie
Laurent	LEFEBVRE	llefebvre@ch-aix.fr	Aix en Provence - SMIT
Johann	AUCHABIE	johann.auchabie@ch-cholet.fr	Chollet - Réanimation
Roxane	COURTOIS	roxane.courtois@ch-cholet.fr	Chollet - SMIT
Karine	LACOMBE	karine.lacombe2@aphp.fr	Paris - Saint Antoine - SMIT
Nathalie	DE CASTRO	nathalie.de-castro@aphp.fr	Paris - Saint Louis - Réanimation
Blandine	RAMMAERT	blandine.rammaert@chu-poitiers.fr	Poitiers - SMIT
Jean-Luc	DIEHL	jean-luc.diehl@aphp.fr	Paris - HEGP - Réanimation
Hugues	AUMAÎTRE	hugues.aumaitre@ch-perpignan.fr	Perpignan - SMIT
Grégory	CORVAISIER	gregory.corvaisier@ch-bretagne-atlantique.fr	Vannes - SMIT
Cédric	JOSEPH	joseph.cedric@chu-amiens.fr	Amiens - SMIT/Réanimation
Pierre-Adrien	BOLZE	pierre-adrien.bolze@chu-lyon.fr	Lyon Sud - Obstétrique
Firouzé	BANI-SADR	fbanisadr@chu-reims.fr	Reims - SMIT
Simon	BESSIS	simon.bessis@aphp.fr	Garches - SMIT
Hajnal-Gabriela	ILLES	gabriela.illes@ch-mdm.fr	Mont de Marsan - SMIT
Antoine	MERCKX	antoine.merckx@ch-cahors.fr	Cahors - SMIT

Younes	KERROUMI	ykerroumi@hopital-dcss.org	Diaconesses CSS - Médecine interne
Cyril	LE BRIS	cyril.le-bris@ch-beziers.fr	Beziers - SMIT/Réanimation
Brigitte	ELHARRAR	Brigitte.elharrar@chicreteil.fr	Créteil CHIC - Médecine interne
Nathalie	ALLOU	nathalie.allou@chu-reunion.fr	Saint Denis - Saint Pierre - SMIT
Corinne	DANIEL	c.daniel@chsaintmartin.fr	Saint Martin - Médecine UDSMT
Cécile	FICKO	cecile.ficko@gmail.com	Bégin -SMIT
Benoît	ROZE	b.roze@ch-saintonge.fr	Saintes - Réanimation
Valérie	GABORIEAU	valerie.gaborieau@ch-pau.fr	Pau - SMIT/Réanimation
Segolene	GREFFE	segolene.greffe@aphp.fr	Boulogne Billancourt - A. Paré -Médecine interne
Olivier	LESENS	olesens@chu-clermontferrand.fr	Clermont-Ferrand - SMIT
Jean-Charles	GAGNARD	jeancharles.gagnard@gmail.com	Antony - Médecine interne
Simon-Djamel	THIBERVILLE	thiberville.sd@ch-manosque.fr	Manosque - SMIT
Stanislas	REBAUDET	s.rebaudet@hopital-europeen.fr	Marseille - SMIT
Pauline	CARAUX PAZ	pauline.caraux-paz@chiv.fr	Villeneuve Saint Georges - SMIT
Moïse	MACHADO	mmachado@ghef.fr	Marne la Vallee- SMIT
Olivier	PICONE	olivier.picone@aphp.fr	Gynécologie,Hôpital Louis Mourrier, Colombe
Elsa	NYAMANKOLLY	NYAMANKOLLYe@ch-dax.fr	Dax - SMIT/Réanimation
Jean-Benoit	ZABBE	marion.zabbe@ch-perigueux.fr	Perigueux - SMIT
Camille	BOUISSE	cbouisse@ch-bourg01.fr	Bourg en Bresse - Infectiologie/Réanimation
Ali	HACHEMI	ali.hachemi@ch-soissons.fr	Soissons - Infectiologie
François	BISSUEL	f-bissuel@ch-hopitauxduleman.fr	Thonon les Bains - Pneumologie
Mélanie	RORIZ	Rorizm@ch-agen-nerac.fr	Agen - Médecine Interne

Anne Sophie	RESSEGUIER	annesophie.resseguier@ch-lepuy.fr	Puy en Velay - Médecine interne
Marie	LACOSTE	mlacoste@ch-alpes-leman.fr	Contamine sur Arve - Infectiologie/Réanimation
Anne Sophie	BOUREAU	annesophie.boureau@chu-nantes.fr	Nantes - Gériatrie
Olivier	LAIREZ	lairez.o@chu-toulouse.fr	Toulouse-cardiologie
Laurent	GUILLEMINAULT	guilleminault.l@chu-toulouse.fr	Toulouse Larrey - Pneumologie
Marc	LAMBERT	Marc.LAMBERT@chru-lille.fr	Lille Calmette - SMIT
Hélène	SALVATOR	h.salvator@hopital-foch.org	Suresnes - Hopital Foch - DRCI
Karen	DELAVIGNE	delavigne.karen@iuct-oncopole.fr	Toulouse - Hématologie/Médecine interne
			Lyon - Hôpital Mère Enfant - Gynécologie
Christophe	RAPP	rappchristophe5@gmail.com; christophe.rapp@ahparis.org	Neuilly sur Seine - Médecine Interne
Eric	DELAVEUVE	e.delaveuve@chr-metz-thionville.fr	Thionville - Bel Air - SMIT/Réanimation
Bertrand	DUSSOL	bertrand.dussol@ap-hm.fr	Marseille conception - Néphrologie
Marc	LEONE	marc.leone@ap-hm.fr	Marseille Nord - La Timone - Réanimation
Jeanne	TRUONG	jeanne.truong@aphp.fr	Paris - Robert Debré - Pédiatrie
Hikombo	HITOTO	hhitoto@ch-lemans.fr	Le Mans CH - SMIT
Vincent	Langlois	vincent.langlois@ch-havre.fr,	Le Havre - MI / Pneumologie
Axelle	BRACONNIER	a.braconnier@hotmail.fr	Mayotte - Gynécologie

Co-author 2

Name	***Last Name***	***Mail address @***	***Affiliation***
Tiphaine	GOULENOK	tiphaine.goulenok@aphp.fr	Paris - Bichat - SMIT
Juliette	PATRIER	juliette.patrier@aphp.fr	Paris - Bichat - Réanimation
Thomas	PERPOINT	thomas.perpoint@chu-lyon.fr	Lyon - SMIT
Amandine	GAGNEUX-BRUNON	amandine.gagneux-brunon@chu-st-etienne.fr	Saint Etienne - SMIT
Nicolas	TERZI	nterzi@chu-grenoble.fr	Grenoble - SMIT
Gabriel	MACHEDA	gmacheda@ch-annecygenevois.fr	Annecy - SMIT
Mathieu	BLOT	mathieu.blot@chu-dijon.fr	Dijon - SMIT
Véronique	LEMEE	Veronique.Lemee@chu-rouen.fr	Rouen - SMIT
Saad	NSEIR	saadalla.nseir@chru-lille.fr	Lille - Réanimation
Olivier	ROBINEAU	olivier.robineau82@gmail.com	Tourcoing - SMIT
Fanny	VUOTTO	Fanny.VUOTTO@CHRU-LILLE.FR	Lille - SMIT
Sabelline	BOUCHEZ	Sabelline.BOUCHEZ@chu-nantes.fr	Nantes - SMIT
Céline	MICHELANGELLI	michelangeli.c@chu-nice.fr	Nice - SMIT
Matthieu	LESOUHAITIER	mathieu.LESOUHAITIER@chu-rennes.fr	Rennes - SMIT
Jérémie	PASQUIER	jeremie.pasquier@chu-martinique.fr	Fort de France - SMIT
Bruno	LEVY	b.levy@chru-nancy.fr	Nancy - Réanimation
Christian	RABAUD	c.rabaud@chru-nancy.fr	Nancy - SMIT
Laetitia	BODENES	Laetitia.bodenes@chu-brest.fr	Brest - Réanimation
Nathalie	PANSU	n-pansu@chu-montpellier.fr	Montpellier - SMIT
Pierre	DELOBEL	delobel.p@chu-toulouse.fr	Toulouse - SMIT
Alberto	SOTTO	albert.sotto@chu-nimes.fr	Nimes - SMIT
Nathalie	DOURNON	nathaliedournon@gmail.com	Bobigny - Avicenne - SMIT
Mehdi	MEZIDI	mehdi.mezidi@chu-lyon.fr	Lyon - Réanimation
Mayka	MERGEAYFABRE	mayka.mergeayfabre@ch-cayenne.fr	Cayenne - SMIT/Réanimation
Rostane	GACI	r.gaci@chr-metz-thionville.fr	Metz - Réanimation

Alexandra	DUCANCELLE	alexandra.ducancelle@univ-angers.fr	Angers - SMIT
Kévin	BOUILLER	kbouiller@chu-besancon.fr	Besancon - SMIT
Romain	DECOURS	romain.decours@chd-vendee.fr	La Roche Sur Yon - Infectiologie
Laurent	PLANTIER	laurent.plantier@univ-tours.fr	Tours - Réanimation
Stéphane	JAUREGUIBERRY	stephane.jaureguiberry@aphp.fr	Kremlin-Bicêtre -SMIT/Médecine interne
Cécile	TROMEUR	cecile.tromeur@chu-brest.fr	Brest - SMIT
Clara	MOUTON PERROT	mouttonperrot.clara@gmail.com	Avignon - SMIT
Carola	PIEROBON	carola.pierobon@ch-metropole-savoie.fr	Chambery - SMIT
Catherine	CHAKVEATZE	eka.chakvetadze@aphp.fr	Melun - SMIT
Rachida	OUISSA	rachida.ouissa@chu-guadeloupe.fr	Guyane - Guadeloupe -Réanimation - SMIT
Laurène	AZEMAR	laurene.azemar@aphp.fr	Paris - Lariboisière - SMIT
Julie	MANKIKIAN	J.MANKIKIAN@chu-tours.fr	Tours - SMIT
Nicolas	CARLIER	nicolas.carlier@aphp.fr	Paris - Cochin - CIC Vaccinologie
Pascal	GRANIER	pgranier@ch-aix.fr	Aix en Provence - SMIT
Anthony	Lemeur	anthony.lemeur@ch-cholet.fr	Chollet - Réanimation
Thibault	CHIARABINI	Thibault.chiarabini@aphp.fr	Paris - Saint Antoine - SMIT
Geoffrey	LIEGEON	geoffroy.liegeon@aphp.fr	Paris - Saint Louis - Réanimation
Gwenaël	Le Moal	Gwenaël.LEMOAL@chu-poitiers.fr	Poitiers - SMIT
Marine	LIVROZET	marine.livrozet@aphp.fr	Paris - HEGP - Réanimation
Delphine	LARIVIERE	delphine.lariviere@ch-bretagne-atlantique.fr	Vannes - SMIT
Julien	MOYET	moyet.julien@chu-amiens.fr	Amiens - SMIT/Réanimation
Maxime	HENTZIEN	mhentzien@chu-reims.fr	Reims - SMIT
Bouchra	LOUTFI	bouchra.loutfi@ch-mdm.fr	Mont de Marsan - SMIT
Vanina	MEYSSONNIER	vmeyssonnier@hopital-dcss.org	Diaconesses CSS - Médecine interne

Benoit	THILL	benoit.thill@ch-beziers.fr	Beziers - SMIT/Réanimation
Valerie	GARRAIT	valerie.garrait@chicreteil.fr	Créteil CHIC - Médecine interne
Marie	LAGRANGE	marie.lagrange-xelot@chu-reunion.fr	Saint Denis - Saint Pierre - SMIT
		-	
Marie	GOMINET	marie.gominet@intradef.gouv.fr	Bégin -SMIT
Delphine	BREGEAUD	d.bregeaud@ch-saintonge.fr	Saintes - Réanimation
Eve	LE COUSTUMIER	eve.lecoustumier@ch-pau.fr	Pau - SMIT/Réanimation
Frédérique	RETORNAZ	f.retornaz@hopital-europeen.fr	Marseille - SMIT
Laurent	RICHIER	laurent.richier@aphp.fr	Villeneuve Saint Georges - SMIT
Audrey	BARRELET	abarrelet@ghef.fr	Marne la Vallee- SMIT
Jeanne	SIBIUDE	Jeanne.sibiude@aphp.fr	Colombes - Louis Mourier - Gynécologie
Adrien	AUVET	auveta@ch-dax.fr	Dax - SMIT/Réanimation
Florent	PEELMAN	florent.peelman@ch-perigueux.fr	Perigueux - SMIT
Nicholas	SEDILLOT	nsedillot@ch-bourg01.fr	Bourg en Bresse - Infectiologie/Réanimation
Patrick	RISPAL	rispalp@ch-agen-nerac.fr	Agen - Médecine Interne
Marlène	MURRIS	murris.m@chu-toulouse.fr	Toulouse Larrey - Pneumologie
Arnaud	SCHERPEREEL	Arnaud.SCHERPEREEL@chru-lille.fr	Lille Calmette - SMIT
Erwan	FOURN	e.fourn@hopital-foch.org	Suresnes - Hopital Foch - DRCI
Stéphane	LASRY	stephane.lasry@ahparis.org	Neuilly sur Seine - Médecine Interne
Coline	JAUD-FISCHER	C.JAUDFISCHER@chru-nancy.fr	Thionville - Bel Air - SMIT/Réanimation
Bruno	PASTENE	bruno.pastene@ap-hm.hm	Marseille Nord - La Timone - Réanimation
Laure	GOUBERT	laure.goubert@ch-havre.fr	Le Havre - MI / Pneumologie

Co-author 3			
Name	***Last name***	***Mail address @***	***Affiliation***
Dominique	LUTON	dominique.luton@aphp.fr	Paris - Bichat - SMIT
Paul Henri	WICKY	paul-henri.wicky@aphp.fr	Paris - Bichat - Réanimation
Anne	CONRAD	anne.conrad@chu-lyon.fr	Lyon - SMIT
Tiffany	TROUILLON	tiffany.trouillon@chu-st-etienne.fr	Saint Etienne - SMIT
Jean-François	PAYEN	JFPayen@chu-grenoble.fr	Grenoble - SMIT
Mylène	MAILLET	mmaillet@ch-annecygenevois.fr	Annecy - SMIT
Sophie	MAHY	sophie.mahy@chu-dijon.fr	Dijon - SMIT
Eglantine	FERRAND DEVOUGE	E.Ferrand-Devouge@chu-rouen.fr	Rouen - SMIT
Sébastien	PREAU	sebastien.preau@chru-lille.fr	Lille - Réanimation
Agnès	MEYBECK	ameybeck@ch-tourcoing.fr	Tourcoing - SMIT
Marie-Charlotte	CHOPIN	Mariecharlotte.CHOPIN@CHRU-LILLE.FR	Lille - SMIT
Romain	GUERY	dr.guery@groupeconfluent.fr	Nantes - SMIT
Karine	RISSO	risso.k@chu-nice.fr	Nice - SMIT
Matthieu	REVEST	matthieu.revest@chu-rennes.fr	Rennes - SMIT
André	CABIE	andre.cabie@chu-martinique.fr	Fort de France - SMIT
Maximilen	SAINT GILLES	M.SAINTGILLES@chru-nancy.fr	Nancy - Réanimation
Sibylle	BEVILACQUA	s.bevilacqua@chru-nancy.fr	Nancy - SMIT
Nicolas	FERRIERE	nicoferriere@yahoo.fr	Brest - Réanimation
Clément	LE BIHAN	c-lebihan@chu-montpellier.fr	Montpellier - SMIT
Benjamine	SARTON	sarton.b@chu-toulouse.fr	Toulouse - SMIT
Didier	Laureillard	didier.laureillard@chu-nimes.fr	Nimes - SMIT
Olivier	BOUCHAUD	olivier.bouchaud@aphp.fr	Bobigny - Avicenne - SMIT
Hodane	YONIS	hodane.yonis@chu-lyon.fr	Lyon - Réanimation

Arsène	KPANGON	amadohoue.kpangon@ch-cayenne.fr	Cayenne - SMIT/Réanimation
Nadia	OUAMARA	n.ouamara@chr-metz-thionville.fr	Metz - Réanimation
Vincent	DUBEE	vincent.dubee@chu-angers.fr	Angers - SMIT
Maïder	PAGADOY	mpagadoy@chu-besancon.fr	Besancon - SMIT
Thomas	GUIMARD	thomas.guimard@chd-vendee.fr	La Roche Sur Yon - Infectiologie
Valérie	GISSOT	valerie.gissot@univ-tours.fr	Tours - Réanimation
Antoine	CHERET	antoine.cheret@aphp.fr	Kremlin-Bicêtre -SMIT/Médecine interne
Dewi	GUELLEC	dewi.guellec@chu-brest.fr	Brest - SMIT
Vincent	PESTRE	PESTRE.Vincent@ch-avignon.fr	Avignon - SMIT
Marie-Christine	CARRET	mariechristine.carret@ch-metropole-savoie.fr	Chambery - SMIT
Clara	FLATEAU	clara.flateau@ghsif.fr	Melun - SMIT
Isabelle	FABRE	mfabre@ghnd.fr	Guyane - Guadeloupe -Réanimation - SMIT
Guylaine	CASTOR-ALEXANDRE	guylaine.alexandre@aphp.fr	Paris - Lariboisière - SMIT
Thomas	FLAMENT	T.FLAMENT@chu-tours.fr	Tours - SMIT
Liem	LUONG	liem.luong@aphp.fr	Paris - Cochin - CIC Vaccinologie
Laurence	MAULIN	lmaulin@ch-aix.fr	Aix en Provence - SMIT
Thierry	MAZZONI	thierry.mazzoni@ch-cholet.fr	Chollet - Réanimation
Bénédicte	LEFEBVRE	benedicte.lefebvre2@aphp.fr	Paris - Saint Antoine - SMIT
Diane	PONSCARME	diane.ponscarme@aphp.fr	Paris - Saint Louis - Réanimation
Isabelle	PIRONNEAU	Isabelle.PIRONNEAU@chu-poitiers.fr	Poitiers - SMIT
Bernard	CHOLLEY	bernard.cholley@aphp.fr	Paris - HEGP - Réanimation
Marie	LANGELOT-RICHARD	marie.langelot-richard@ch-bretagne-atlantique.fr	Vannes - SMIT
Cinthia	RAMES	rames.cinthia@chu-amiens.fr	Amiens - SMIT/Réanimation
Yohan	N'GUYEN	yohan.nguyen@wanadoo.fr<br>ynguyen@chu-reims.fr	Reims - SMIT

Jérôme	DIMET	jerome.dimet@ght40.fr	Mont de Marsan - SMIT
Oryane	MABIALA	omabiala@for.paris	Diaconesses CSS - Médecine interne
Marie-Laure	CASANOVA	marie-laure.casanova@ch-beziers.fr	Beziers - SMIT/Réanimation
Isabelle	DELACROIX	isabelle.delacroix@chicreteil.fr	Créteil CHIC - Médecine interne
Julien	JABOT	jabot974@gmail.com	Saint Denis - Saint Pierre - SMIT
Aurore	BOUSQUET	aurorebousquet@yahoo.fr	Bégin -SMIT
Younes	AIT TAMLIHAT	y.ait-tamlihat@ch-saintonge.fr	Saintes - Réanimation
Walter	PICARD	walter.picard@ch-pau.fr	Pau - SMIT/Réanimation
Myriam / Hortense	BENNANI / DROUET	M.BENNANI@hopital-europeen.fr / h.drouet@hopital-europeen.fr	Marseille - SMIT
Danielle	JAAFAR	danielle.jaafar@chiv.fr	Villeneuve Saint Georges - SMIT
Alexandra	BEDOSSA	abedossa@ghef.fr	Marne la Vallee- SMIT
Laurent	MANDELBROT	laurent.mandelbrot@aphp.fr	Colombes - Louis Mourier - Gynécologie
Anne-Hélène	BOIVIN	helene.boivin@ght40.fr	Dax - SMIT/Réanimation
Edouard	SOUM	edouard.soum@ch-perigueux.fr	Perigueux - SMIT
Damien	BOUHOUR	dbouhour@ch-bourg01.fr	Bourg en Bresse - Infectiologie/Réanimation
Sarah	REDL	redls@ch-agen-nerac.fr	Agen - Médecine Interne
Agnès	SOMMET	agnes.sommet@univ-tlse3.fr	Toulouse Larrey - Pneumologie
Ryadh	POKEERBUX	ryadh.pokeerbux@chru-lille.fr	Lille Calmette - SMIT
David	ZUCMAN	d.zucman@hopital-foch.org	Suresnes - Hopital Foch - DRCI
Thierry	CARMOI	thierry.carmoi@ahparis.org	Neuilly sur Seine - Médecine Interne
Paul	DUNAND	paul.m.dunand@gmail.com	Thionville - Bel Air - SMIT/Réanimation
Karine	BEZULIER	karine.bezulier@ap-hm.fr	Marseille Nord - La Timone - Réanimation
Stéphanie	COUSSE	stephanie.cousse@ch-havre.fr	Le Havre - MI / Pneumologie

Co-author 4			
Name	***Last name***	***Mail address @***	***Affiliation***
Lauren	DECONINCK	bastien.deconninck@aphp.fr	Paris - Bichat - SMIT
Lucie	LE FEVRE	lucie.lefevre@aphp.fr	Paris - Bichat - Réanimation
Laurence	BOUILLET	lbouillet@chu-grenoble.fr	Grenoble - SMIT
Patrick	IMBERT	pimbert@ch-annecygenevois.fr	Annecy - SMIT
Marielle	BUISSON	marielle.buisson@chu-dijon.fr	Dijon - SMIT
Kévin	ALEXANDRE	kevin.alexandre@chu-rouen.fr	Rouen - SMIT
Mercé	JOURDAIN	merce.jourdain@chru-lille.fr	Lille - Réanimation
Sarah	STABLER	stabler.sarah@gmail.com	Lille - SMIT
Paul	LE TURNIER	Paul.LETURNIER@chu-nantes.fr	Nantes - SMIT
Pierre	TATTEVIN	pierre.tattevin@chu-rennes.fr	Rennes - SMIT
Pierre-François	SANDRINE	sandrine.pierre-francois@chu-martinique.fr	Fort de France - SMIT
Benjamin	LEFEVRE	B.LEFEVRE@chru-nancy.fr	Nancy - SMIT
Stella	Rousset	rousset.st@chu-toulouse.fr	Toulouse - SMIT
Guillaume	LOUIS	g.louis@chr-metz-thionville.fr	Metz - Réanimation
Quentin	LEPILLER	q1lepiller@chu-besancon.fr	Besancon - SMIT
Emmanuelle	MERCIER	emercier@med.univ-tours.fr	Tours - Réanimation
Florence	JEGO	florence.jego@ch-metropole-savoie.fr	Chambery - SMIT
Pierre-Marie	ROGER	pierre-marie.roger@chu-guadeloupe.fr	Guyane - Guadeloupe -Réanimation - SMIT
Marie	LACHATRE	marie.lachatre@aphp.fr	Paris - Cochin - CIC Vaccinologie
Jean-Benoit	ARLET	jean-benoit.arlet@aphp.mssante.fr	Paris - HEGP - Réanimation
Juliette	ROMARU	jromaru@chu-reims.fr	Reims - SMIT
Georges	LE FALHER	georges.le-falher@ch-beziers.fr	Beziers - SMIT/Réanimation
Thomas	MAITRE	thomas.maitre@chicreteil.fr	Créteil CHIC - Médecine interne

Claudine	BADR	claudine.Badr@chiv.fr	Villeneuve Saint Georges - SMIT
Stéphanie	FRY	Stephanie.FRY@chru-lille.fr	Lille Calmette - SMIT
Marie-Laure	CHABI-CHAVILLAT	ml.chabi-charvillat@hopital-foch.com	Suresnes - Hopital Foch - DRCI

Co-author 5			
Name	***Last name***	***Mail adress @***	***Affiliation***
Sylvie	LE GAC	sylvie.legac@aphp.fr	Paris - Bichat - SMIT
Pierre	JACQUET	Pierre.jaquet@aphp.fr	Paris - Bichat - Réanimation
Rebecca<br>Marion	HAMIDFAR<br>LE MARECHAL	rhamidfar@chu-grenoble.fr;<br>mlemarechal@chu-grenoble.fr	Grenoble - SMIT<br>Grenoble - SMIT
Amélie	VALRAN	avalran@ch-annecygenevois.fr	Annecy - SMIT
Lionel	PIROTH	lionel.piroth@chu-dijon.fr	Dijon - SMIT
Elise	ARTAUD-MACCARI	Elise.Artaud-Macari@chu-rouen.fr	Rouen - SMIT
Raphaël	FAVORY	raphael.favory@chru-lille.fr	Lille - Réanimation
Jules	BAUER	bauerjules@gmail.com	Lille - SMIT
Cécile	MEAR-PASSARD	cecile.passard@chu-nantes.fr	Nantes - SMIT
Jean-Marc	CHAPPLAIN	jean-marc.chapplain@chu-rennes.fr	Rennes - SMIT
Jean-Marie	TURMEL	jean-marie.turmel@chu-martinique.fr	Fort de France - SMIT
Anne<br>Guillaume	GUILLAUMOT<br>MARTIN-BLONDEL	a.guillaumot@chru-nancy.fr<br>martin-blondel.g@chu-toulouse.fr	Nancy - SMIT<br>Toulouse - SMIT
Cyril	CADOZ	c.cadoz@chr-metz-thionville.fr	Metz - Réanimation
Noémie	TISSOT	noemie.tissot@univ-fcomte.fr	Besancon - SMIT
Charlotte	SALMON<br>GANDONNIERE	charlotte.salmon.gandonniere@gmail.com	Tours - Réanimation
Margaux	ISNARD	margaux.isnard@ch-metropole-savoie.fr	Chambery - SMIT
Samuel	Markowicz	samuel.markowicz@chu-guadeloupe.fr	Guyane - Guadeloupe -Réanimation - SMIT
Odile	LAUNAY	odile.launay@aphp.fr	Paris - Cochin - CIC Vaccinologie
Olivier	SANCHEZ	manuel.sanchez@aphp.fr	Paris - HEGP - Réanimation
Kévin	DIDIER	kdidier@chu-reims.fr	Reims - SMIT
Eric	OZIOL	eric.oziol@ch-beziers.fr	Beziers - SMIT/Réanimation
Jean Baptiste	ASSIE	jean-baptiste.assie@inserm.fr	Créteil CHIC - Médecine interne

Fara	DIOP	Fara.Diop@chiv.fr	Villeneuve Saint Georges - SMIT
Cécile	YELNIK	CECILE.YELNIK@chru-lille.fr	Lille Calmette - SMIT
Aurélie	MARTIN	a.martin@hopital-foch.com	Suresnes - Hopital Foch - DRCI
