# Supplementary material for: Modeling SARS-CoV-2 viral kinetics and association with mortality in hospitalized patients from the French COVID cohort
Source: Proc Natl Acad Sci U S A. 2021 Feb 3;118(8):e2017962118. doi: 10.1073/pnas.2017962118 (PMC7929555; doi:10.1073/pnas.2017962118)
Supplement: Supplementary File [file pnas.2017962118.sapp01.pdf]

Name	***Last name***	***Mail address @***	***Affiliation***
Laurent	ABEL	laurent.abel@inserm.fr	Inserm UMR 1163, Paris, France
Claire	ANDREJAK	andrejak.claire@chu-amiens.fr	CHU Amiens, France
François	ANGOULVANT	francois.angoulvant@aphp.fr	Hôpital Necker, Paris, France
Delphine	BACHELET	delphine.bachelet@aphp.fr	Hôpital Bichat, Paris, France
Marie	BARTOLI	marie.bartoli@anrs.fr	ANRS, Paris, France
Romain	BASMACI	romain.basmaci@aphp.fr	Hôpital Louis Mourier, Colombes, France
Sylvie	BEHILILL	sylvie.behillil@pasteur.fr	Pasteur Institute, Paris, France
Marine	BELUZE	marine.beluze@aphp.fr	F-CRIN Partners Platform, Paris, France
Dehbia	BENKERROU	dehbia.benkerrou@iplesp.upmc.fr	Inserm UMR 1136, Paris, France
Krishna	BHAVSAR	krishna.bhavsar@aphp.fr	Hôpital Bichat, Paris, France
François	BOMPART	fbompart@dndi.org	Drugs for Neglected Diseases initiative, Geneva, Switzerland
Lila	BOUADMA	lila.bouadma@aphp.fr	Hôpital Bichat, Paris, France
Maude	BOUSCAMBERT	maude.bouscambert-duchamp@chu-lyon.fr	Inserm UMR 1111, Lyon, France
Minerva	CERVANTES-GONZALEZ	minerva.cervantes@inserm.fr	REACTing, Paris, France
Anissa	CHAIR	anissa.chair@aphp.fr	Hôpital Bichat, Paris, France
Catherine	CHIROUZE	catherine.chirouze@univ-fcomte.fr	CHRU Jean Minjoz, Besançon, France
Alexandra	COELHO	alexandra.coelho@inserm.fr	Inserm UMR 1018, Paris, France
Sandrine	COUFFIN-CADIERGUES	sandrine.couffin-cadiergues@inserm.fr	Inserm sponsor, Paris, France
Camille	COUFFIGNAL	camille.couffignal@aphp.fr	Hôpital Bichat, Paris, France

Eric	d'ORTENZIO	eric.dortenzio@inserm.fr	REACTing, Paris, France
Charlene	DA SILVEIRA	charlene.dasilveira@aphp.fr	Hôpital Bichat, Paris, France
Marie-Pierre	DEBRAY	marie-pierre.debray@aphp.fr	Hôpital Bichat, Paris, France
Dominique	DEPLANQUE	Dominique.DEPLANQUE@chru-lille.fr	Hôpital Calmette, Lille, France
Diane	DESCAMPS	diane.descamps@aphp.fr	Hôpital Bichat, Paris, France
Mathilde	DESVALLÉE	mathilde.desvallees@u-bordeaux.fr	Inserm UMR 1219, Bordeaux, France
Alpha	DIALLO	alpha.diallo@inserm.fr	ANRS, Paris, France
Alphonsine	DIOUF	alphonsine.diouf@inserm.fr	Inserm UMR 1018, Paris, France
Céline	DORIVAL	celine.dorival@iplesp.upmc.fr	Inserm UMR 1136, Paris, France
François	DUBOS	Francois.DUBOS@CHRU-LILLE.FR	CHU Lille, France
Xavier	DUVAL	xavier.duval@aphp.fr	Hôpital Bichat, Paris, France
Philippine	ELOY	philippine.eloy@aphp.fr	Hôpital Bichat, Paris, France
Vincent	ENOUF	vincent.enouf@pasteur.fr	Pasteur Institute, Paris, France
Hélène	ESPEROU	helene.esperou@inserm.fr	Inserm sponsor, Paris, France
Marina	ESPOSITO-FARESE	marina.esposito-farese@aphp.fr	Hôpital Bichat, Paris, France
Manuel	ETIENNE	Manuel.Etienne@chu-rouen.fr	CHU Rouen, France
Nadia	ETTALHAOUI	nadia.ettalhaoui@aphp.fr	Hôpital Bichat, Paris, France
Nathalie	GAULT	nathalie.gault@aphp.fr	Hôpital Bichat, Paris, France
Alexandre	GAYMARD	alexandre.gaymard@chu-lyon.fr	Inserm UMR 1111, Lyon, France
Jade	GHOSN	jade.ghosn@aphp.fr	Hôpital Bichat, Paris, France

Tristan	GIGANTE	T.GIGANTE@chru-nancy.fr	F-CRIN INI-CRCT, Nancy, France
Morgane	GILG	M.GILG@chru-nancy.fr	F-CRIN INI-CRCT, Nancy, France
Isabelle	GORENNE	isabelle.gorenne@aphp.fr	Hôpital Bichat, Paris, France
Jérémie	GUEDJ	jeremie.guedj@inserm.fr	Inserm UMR 1137, Paris, France
Alexandre	HOCTIN	alexandre.hoctin@inserm.fr	Inserm UMR 1018, Paris, France
Ikram	HOUAS	ikram.houas@inserm.fr	Inserm sponsor, Paris, France
Isabelle	HOFFMANN	isabelle.hoffmann@aphp.fr	Hôpital Bichat, Paris, France
Jean-Sébastien	HULOT	jean-sebastien.hulot@aphp.fr	Hôpital Européen Georges Pompidou, Paris, France
Salma	JAAFOURA	salma.jaafoura@inserm.fr	Inserm sponsor, Paris, France
Ouifiya	KAFIF	ouifiya.kafif@aphp.fr	Hôpital Bichat, Paris, France
Florentia	KAGUELIDOU	florentia.kaguelidou@aphp.fr	Hôpital Robert Debré, Paris, France
Sabrina	KALI	sabrina.kali@aphp.fr	Hôpital Bichat, Paris, France
Antoine	KHALIL	antoine.khalil@aphp.fr	Hôpital Bichat, Paris, France
Coralie	KHAN	coralie.khan@u-bordeaux.fr	Inserm UMR 1219, Bordeaux, France
Cédric	LAOUÉNAN	cedric.laouenan@aphp.fr	Hôpital Bichat, Paris, France
Samira	LARIBI	samira.laribi@aphp.fr	Hôpital Bichat, Paris, France
Minh	LE	minh.le@aphp.fr	Hôpital Bichat, Paris, France
Quentin	LE HINGRAT	quentin.lehingrat@aphp.fr	Hôpital Bichat, Paris, France
Hervé	LE NAGARD	herve.lenagard@inserm.fr	Inserm UMR 1137, Paris, France
Soizic	LE MESTRE	soizic.le mestre@inserm.fr	ANRS, Paris, France
François-	LESCURE	xavier.lescure@aphp.fr	Hôpital Bichat, Paris, France

Xavier			
Yves	LEVY	yves.levy@inserm.fr	Vaccine Research Insitute (VRI), Inserm UMR 955, Créteil, France
Claire	LEVY-MARCHAL	claire.levy-marchal@inserm.fr	F-CRIN INI-CRCT, Paris, France
Bruno	LINA	bruno.lina@chu-lyon.fr	Inserm UMR 1111, Lyon, France
Guillaume	LINGAS	guillaume.lingas@inserm.fr	Inserm UMR 1137, Paris, France
Jean Christophe	LUCET	jean-christophe.lucet@aphp.fr	Hôpital Bichat, Paris, France
Denis	MALVY	denis.malvy@chu-bordeaux.fr	CHU Bordeaux, France
Marina	MAMBERT	marina.mambert@inserm.fr	Inserm UMR 1018, Paris, France
France	MENTRÉ	france.mentre@inserm.fr	Hôpital Bichat, Paris, France
Noémie	MERCIER	noemie.mercier@inserm.fr	ANRS, Paris, France
Amina	MEZIANE	amina.meziane@iplesp.upmc.fr	Inserm UMR 1136, Paris, France
Hugo	MOUQUET	hugo.mouquet@pasteur.fr	Pasteur Institute, Paris, France
Jimmy	Mullaert	jimmy.mullaert@inserm.fr	Hôpital Bichat, Paris, France
Nadège	NEANT	nadege.neant@inserm.fr	Inserm UMR 1137, Paris, France
Marion	NORET	mnoret@ch-annecygenevois.fr	RENARCI, Annecy, France
Justine	PAGES	justine.pages@aphp.fr	Hôpital Robert Debré, Paris, France
Aurélie	PAPADOPOULOS	aurelie.papadopoulos@inserm.fr	Inserm sponsor, Paris, France
Christelle	PAUL	christelle.paul@inserm.fr	ANRS, Paris, France
Nathan	PEIFFER-SMADJA	nathan.peiffer-smadja@inserm.fr	Hôpital Bichat, Paris, France
Ventzislava	PETROV-SANCHEZ	ventzislava.petrov-sanchez@anrs.fr	ANRS, Paris, France

Gilles	PEYTAVIN	gilles.peytavin@aphp.fr	Hôpital Bichat, Paris, France
Olivier	PICONE	olivier.picone@aphp.fr	Hôpital Louis Mourier, Colombes, France
Oriane	PUÉCHAL	oriane.puechal@inserm.fr	REACTing, Paris, France
Manuel	ROSA-CALATRAVA	manuel.rosa-calatrava@univ-lyon1.fr	Inserm UMR 1111, Lyon, France
Bénédicte	ROSSIGNOL	B.ROSSIGNOL@chru-nancy.fr	F-CRIN INI-CRCT, Nancy, France
Patrick	ROSSIGNOL	p.rossignol@chru-nancy.fr	CHU Nancy, France
Carine	ROY	carine.roy@aphp.fr	Hôpital Bichat, Paris, France
Marion	SCHNEIDER	marion.schneider2@aphp.fr	Hôpital Bichat, Paris, France
Caroline	SEMAILLE	caroline.semaille@anses.fr	REACTing, Paris, France
Nassima	SI MOHAMMED	nassima.simohammed@aphp.fr	Hôpital Bichat, Paris, France
Lysa	TAGHERSET	lysa.tagherset@aphp.fr	Hôpital Bichat, Paris, France
Coralie	TARDIVON	coralie.tardivon@aphp.fr	Hôpital Bichat, Paris, France
Marie-Capucine	TELLIER	marie-capucine.tellier@aphp.fr	Hôpital Bichat, Paris, France
François	TÉOULÉ	francois.teoule@iplesp.upmc.fr	Inserm UMR 1136, Paris, France
Olivier	TERRIER	olivier.terrier@univ-lyon1.fr	Inserm UMR 1111, Lyon, France
Jean-François	TIMSIT	jean-francois.timsit@aphp.fr	Hôpital Bichat, Paris, France
Théo	TRIOUX	theo.trioux@aphp.fr	Hôpital Bichat, Paris, France
Christelle	TUAL	christelle.tual@chu-rennes.fr	Inserm CIC-1414, Rennes, France
Sarah	TUBIANA	sarah.tubiana@aphp.fr	Hôpital Bichat, Paris, France
Sylvie	VAN DER WERF	sylvie.van-der-werf@pasteur.fr	Pasteur Institute, Paris, France

Noémie	VANEL	Noemie.VANEL@ap-hm.fr	Hôpital la Timone, Marseille, France
Aurélie	VEISLINGER	Aurelie.VEISLINGER@chu-rennes.fr	Inserm CIC-1414, Rennes, France
Benoit	VISSEAUX	benoit.visseaux@aphp.fr	Hôpital Bichat, Paris, France
Aurélie	WIEDEMANN	aurelie.wiedemann@inserm.fr	Vaccine Research Insitute (VRI), Inserm UMR 955, Créteil, France
Yazdan	YAZDANPANAH	yazdan.yazdanpanah@aphp.fr	Hôpital Bichat, Paris, France
