# Supplementary material for: Modeling SARS-CoV-2 viral kinetics and association with mortality in hospitalized patients from the French COVID cohort
Source: Proc Natl Acad Sci U S A. 2021 Feb 3;118(8):e2017962118. doi: 10.1073/pnas.2017962118 (PMC7929555; doi:10.1073/pnas.2017962118)
Supplement: Supplementary File [file pnas.2017962118.sapp02.pdf]

PNAS
www.pnas.org

Supplementary Information for

Modeling SARS-CoV-2 viral kinetics and association with mortality in hospitalized patients from the French Covid cohort

Nadège Néant[1]*, Guillaume Lingas[1]*, Quentin Le Hingrat[1,2]*, Jade Ghosn[1,3], Ilka Engelmann[4], Quentin Lepiller[5], Alexandre Gaymard[6,7], Virginie Ferré[8], Cédric Hartard[9], Jean-Christophe Plantier[10], Vincent Thibault[11], Julien Marlet[12,13], Brigitte Montes[14], Kevin Bouiller[15], François-Xavier Lescure[3], Jean-François Timsit[16], Emmanuel Faure[17], Julien Poissy[18], Christian Chidiac[19], François Raffi[20,21], Antoine Kimmoun[22], Manuel Etienne[23], Jean-Christophe Richard[24], Pierre Tattevin[25], Denis Garot[26], Vincent Le Moing[27], Delphine Bachelet[28], Coralie Tardivon[28], Xavier Duval[1,29], Yazdan Yazdanpanah[1,3], France Mentré[1,28], Cédric Laouénan[1,28]*, Benoit Visseaux[1,2]*, Jérémie Guedj[1]*, for the French COVID cohort investigators and study group

*: the authors equally contributed

Corresponding author: Nadège Néant
nadege.neant@inserm.fr

This PDF file includes:

- Supplementary text
- Figures S1 to S10 (not allowed for Brief Reports)
- Tables S1 to S8 (not allowed for Brief Reports)
- List of authors of the French Cohort Study group
- List of authors of the French Cohort Investigators group

Supplementary Information Text

1) Data description

A- Viral load data

Ct results were transformed into log_{10} RNA copies/mL using the relationship assessed by Pasteur Institute for both E and RdRp genes [1].

In order to homogenize viral loads after transformations from Ct to log_{10} RNA copies/mL, gene E log_{10} viral loads were converted to IP4 log_{10} viral loads, using regression analysis coefficients determined on patients whose RT-PCR for both genes had been performed ($N=72$, $r=0.95$, $p<10^{-5}$).

Thus, the relationship between viral load transformation from E gene viral load to IP4 viral load was:

$$log_{10}\, VL_{IP4} = -2{,}26321 + 1{,}1211 \times \left(VL_{Egene}\right)$$

B- Lower respiratory tract (LRT) samples

LRT samples were available in 98 patients, and included tracheal aspirate, broncho-alveolar lavage and bronchial aspirate (see Table S1). We compared the mortality in patients for which at least one LRT was available and those for which no LRT data was available (Fig. S9). Finally we compared the viral load levels in individuals having samples at the same time (+/- 2 days). In individuals having several pairs of observations, only the first one was considered.

C- Viral load and survival

We performed a Kaplan Meier survival analysis to compare the cumulative incidence of mortality among patient with high (≥6 log_{10} copies/mL) and low viral load (<6 log_{10} copies/mL) at different time points, i.e., 3, 7 and 14 days (+/- 2 days) after symptom onset. Log rank testing was used to compare the cumulative incidence of mortality between these two groups.

2) Viral dynamic modeling

A- Mathematical models of antigen-driven immune response

We tested several models with an antigen-driven immune response, noted F. For the sake of comparison, we used models with a similar parameterization and the same number of unknown parameters. In all models we assumed that F was dimensionless and we fixed $d_f=0.4\ d^{-1}$

Model A. Target-cell limited model with an eclipse phase

$$\frac{dT}{dt} = -\beta V_i T$$

$$\frac{dI_1}{dt} = \beta V_i T - kI_1$$

$$\frac{dI_2}{dt} = kI_1 - \delta I_2$$

$$\frac{dV_i}{dt} = p\mu I_2 - cV_i$$

$$\frac{dV_{ni}}{dt} = p(1-\mu)I_2 - cV_{ni}$$

Model B: Immune effector F leads to protection of target cells

$$\frac{dT}{dt} = -\beta V_i T - \ \phi \frac{F}{F+\theta} T$$

$$\frac{dI_1}{dt} = \beta V_i T - kI_1$$

$$\frac{dI_2}{dt} = kI_1 - \delta I_2$$

$$\frac{dV_i}{dt} = p\mu I_2 - cV_i$$

$$\frac{dV_{ni}}{dt} = p(1-\mu)I_2 - cV_{ni}$$

$$\frac{dF}{dt} = I_2 - d_f F$$

Model C: Immune effector F blocks viral production

$$\frac{dT}{dt} = -\beta V_i T$$

$$\frac{dI_1}{dt} = \beta V_i T - kI_1$$

$$\frac{dI_2}{dt} = kI_1 - \delta I_2$$

$$\frac{dV_i}{dt} = p \times \left(1 - \phi \frac{F}{F+\theta}\right) \times \mu I_2 - cV_i$$

$$\frac{dV_{ni}}{dt} = p \times \left(1 - \phi \frac{F}{F + \theta}\right) \times (1 - \mu)I_2 - cV_{ni}$$

$$\frac{dF}{dt} = I_2 - d_f F$$

Model D: Immune effector F blocks cell infection

$$\frac{dT}{dt} = -\beta \times \left(1 - \phi \frac{F}{F + \theta}\right) \times V_i T$$

$$\frac{dI_1}{dt} = \beta \times \left(1 - \phi \frac{F}{F + \theta}\right) \times V_i T - kI_1$$

$$\frac{dI_2}{dt} = kI_1 - \delta I_2$$

$$\frac{dV_i}{dt} = p\mu I_2 - cV_i$$

$$\frac{dV_{ni}}{dt} = p(1 - \mu)I_2 - cV_{ni}$$

$$\frac{dF}{dt} = I_2 - d_f F$$

Model E: Immune effector F increases viral clearance

$$\frac{dT}{dt} = -\beta V_i T$$

$$\frac{dI_1}{dt} = \beta V_i T - kI_1$$

$$\frac{dI_2}{dt} = kI_1 - \delta I_2$$

$$\frac{dV_i}{dt} = p\mu I_2 - cV_i - \phi \frac{F}{F + \theta} V_i$$

$$\frac{dV_{ni}}{dt} = p(1 - \mu)I_2 - cV_{ni} - \phi \frac{F}{F + \theta} V_{ni}$$

$$\frac{dF}{dt} = I_2 - d_f F$$

Model F: Immune effector F increases the elimination of infected cells

$$\frac{dT}{dt} = -\beta V_i T$$

$$\frac{dI_1}{dt} = \beta V_i T - kI_1$$

$$\frac{dI_2}{dt} = kI_1 - \delta I_2 - \phi \frac{F}{F+\theta} I_2$$

$$\frac{dV_i}{dt} = p\mu I_2 - cV_i$$

$$\frac{dV_{ni}}{dt} = p(1-\mu)I_2 - cV_{ni}$$

$$\frac{dF}{dt} = I_2 - d_f F$$

B. Alternative models for the time-dependent change in the elimination rate of infected cells

Model F provided the best fit to the data, suggesting an antigen-dependent effect in the loss rate of infected cells (Table S4).

Next, we tested different other models accounting for the changes in the loss rate of infected cells after day 12, which was the median time of antibody seroconversion. We also considered simpler models where that F and I_2 were in quasi-steady state. However none of these models improved the data fitting (SI appendix, Table S5).

Model F1. Loss rate of productive infected cells takes on a new value after 12 days post symptom onset

$$\frac{dT}{dt} = -\beta V_i T$$

$$\frac{dI_1}{dt} = \beta V_i T - kI_1$$

$$\frac{dI_2}{dt} = kI_1 - \delta I_2$$

$$\frac{dV_i}{dt} = p\mu I_2 - cV_i$$

$$\frac{dV_{ni}}{dt} = p(1-\mu)I_2 - cV_{ni}$$

$$\delta = \delta_0 + \delta_1 I_{\{time>12\}}$$

Model F2. Loss rate of productive infected cells increases continuously after 12 days post symptom onset

The model assumed a saturation after day 30 to reflect the saturation of antibody levels over time

$$\frac{dT}{dt} = -\beta V_i T$$

$$\frac{dI_1}{dt} = \beta V_i T - kI_1$$

$$\frac{dI_2}{dt} = kI_1 - \delta_\tau I_2$$

$$\frac{dV_i}{dt} = p\mu I_2 - cV_i$$

$$\frac{dV_{ni}}{dt} = p(1-\mu)I_2 - cV_{ni}$$

$$\delta_\tau = \delta_0 \; exp^{w\times min\left((t-12),30\right)} I_{\{time>12\}}$$

Model F3: F and I_2 are in quasi steady-state

We studied the case where F and I_2 are in quasi steady state ($F = a \times I_2$), assuming in addition with $\theta \gg F$, leading to a simplification of model F as follows:

$$\frac{dT}{dt} = -\beta V_i T$$

$$\frac{dI_1}{dt} = \beta V_i T - kI_1$$

$$\frac{dI_2}{dt} = kI_1 - \delta I_2 - \phi I_2^2$$

$$\frac{dV_i}{dt} = p\mu I_2 - cV_i$$

$$\frac{dV_{ni}}{dt} = p(1-\mu)I_2 - cV_{ni}$$

Fig. S1. Cumulative incidence of mortality according to the level of viral load at 3 (a), 7 (b) and 14 (c) days since symptom onset.

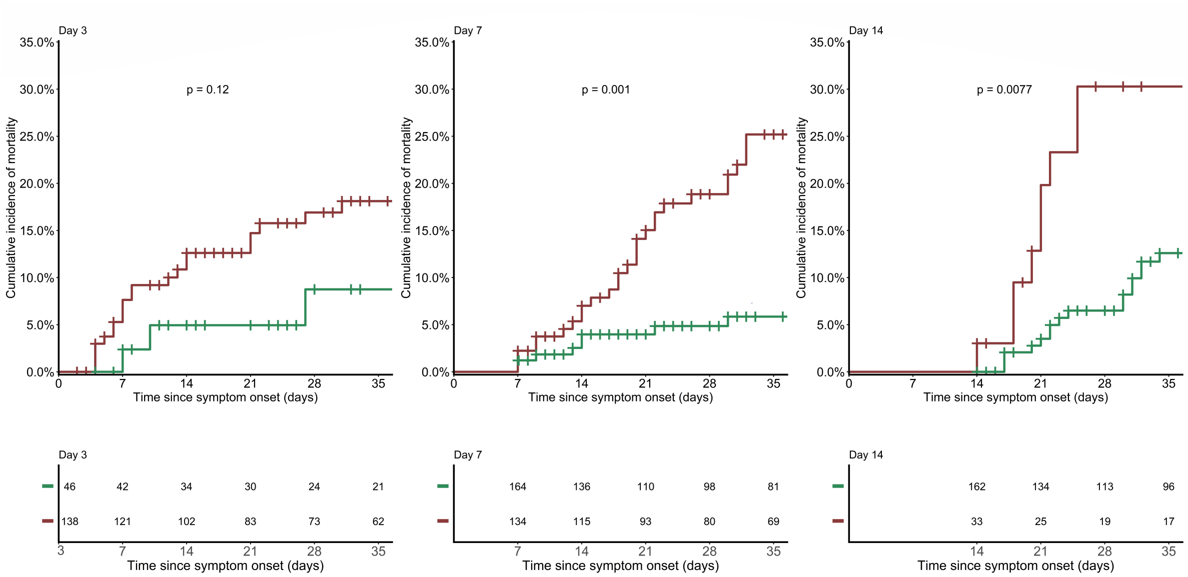

Green line are patient with viral load < 6 $\log_{10}$ copies/mL and red line are patients with viral load ≥ 6 $\log_{10}$ copies/mL at 3, 7 and 14 days post symptom onset (+/- 2 days). Below are the number of patients included in each analysis (first time point) and still available over time.

Fig. S2. The viral kinetic models tested.

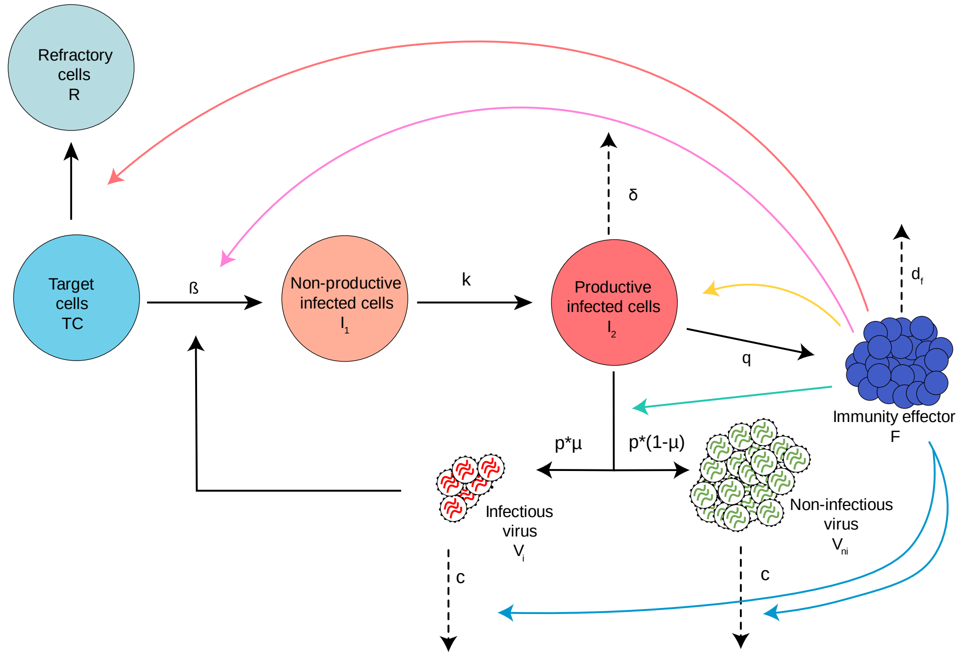

Five models incorporating immune response compartment (F) were compared with the standard model (target-cell limited model): reducing viral infectivity, leading to cells refractory to infection (red arrow), decreasing the rate of viral production (green arrow) and the infection rate (pink arrow), increasing the viral clearance (blue arrow) or the loss rate of infected cell (yellow arrow).

Fig. S3. Individual predictions of nasopharyngeal viral kinetic in the 131 patients for which 3 serial NP samples were available.

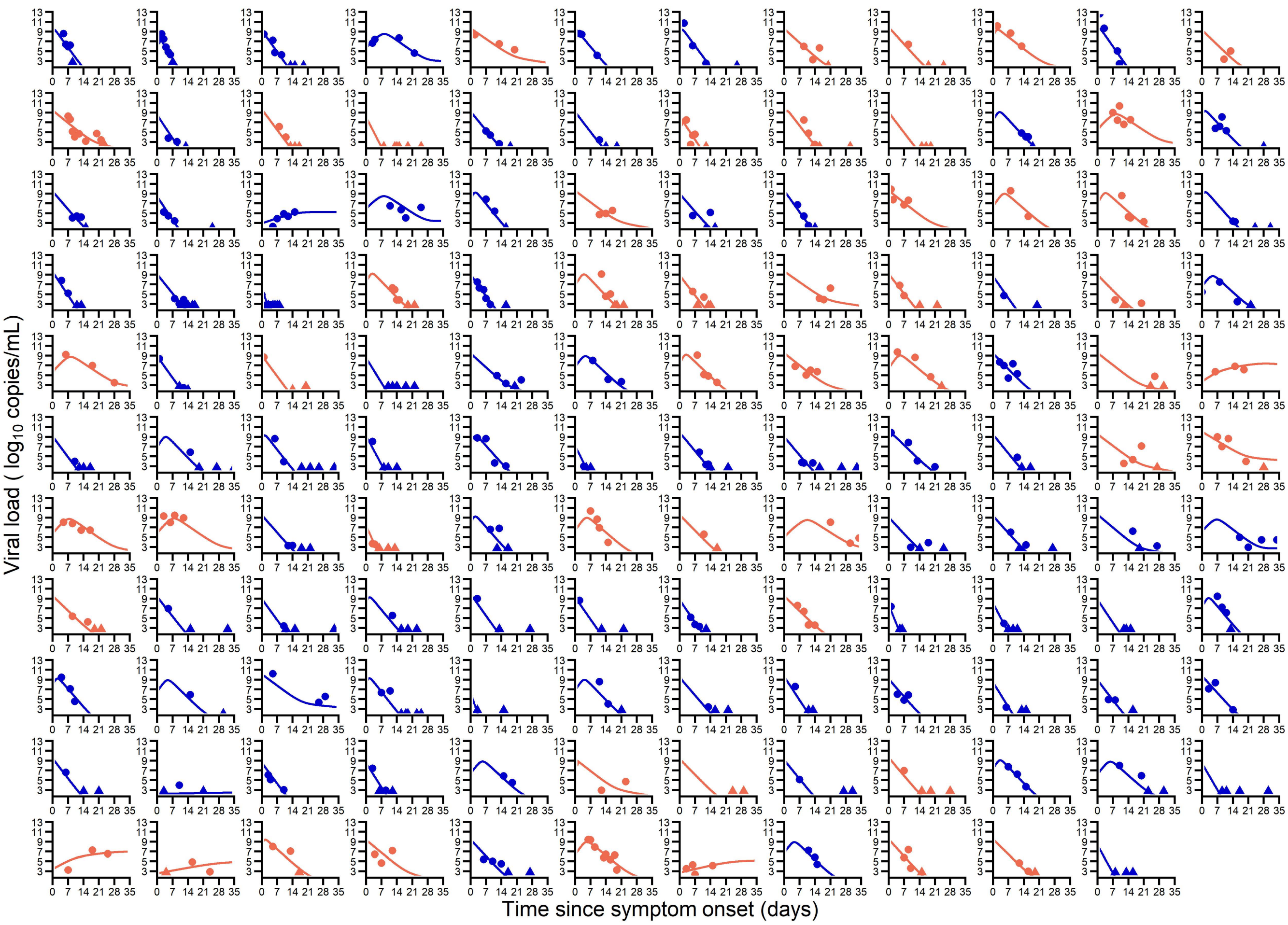

The solid line is the individual prediction of viral load and the circles are the observed data according to age (Blue: age <65 year. Orange: age ≥65). Triangles are data below the limit of detection.

Fig. S4. Visual predictive check of cytotoxic model.

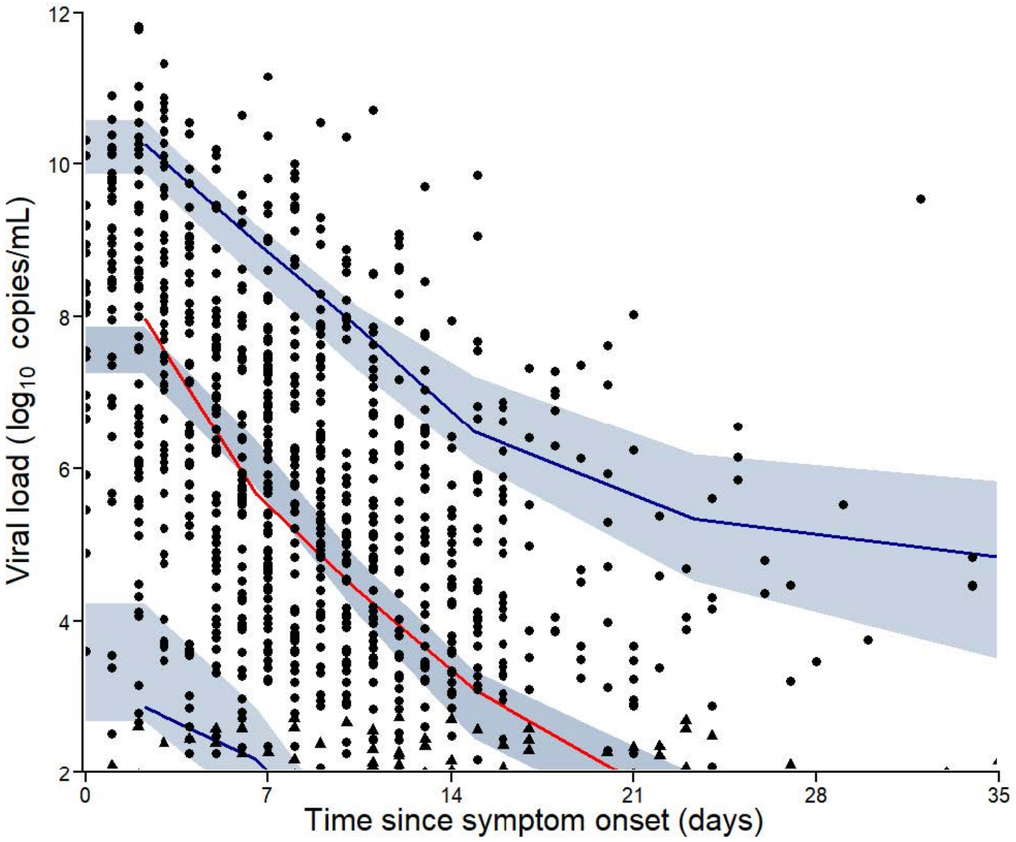

The circles represent the observed data. The triangles represent data below the limit of detection. The red line indicates the 50th percentiles of the observed data. The blue lines indicate the 5th and 95th percentiles of the observed data. The shaded areas represent 90% confidence interval (CI) of the simulated 50th, 5th and 95th percentiles.

Fig. S5. Predicted time to viral clearance according corticosteroid treatment (a) or antiviral treatment (b).

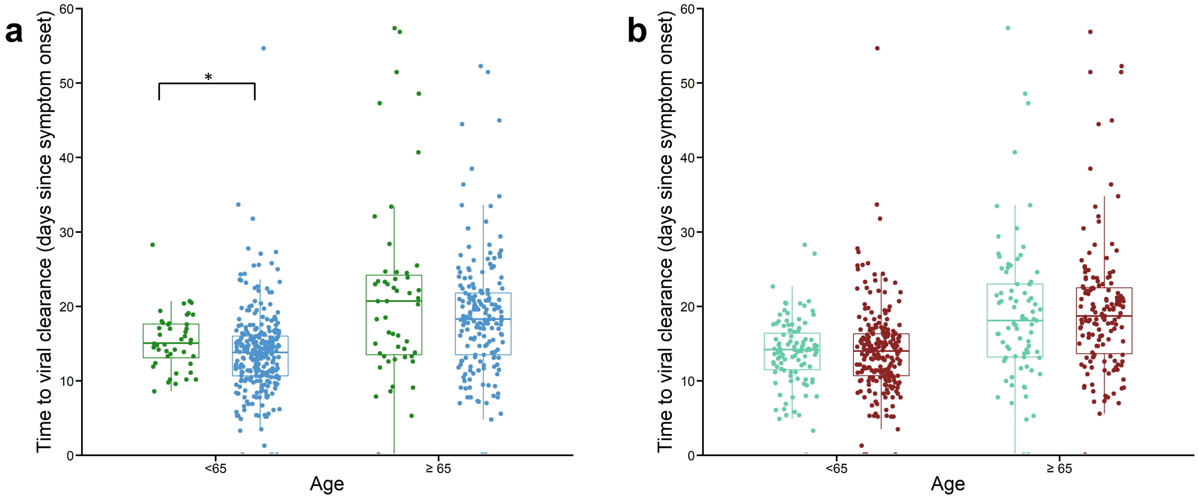

Green: patients treated by corticosteroid, blue: patients not treated by corticosteroid. * Significant difference was found between patients <65 (p=0.01). b) Light green: patients treated by antiviral, brown: patients not treated by antiviral treatment.

Fig. S6. Cumulative incidence of seroconversion IgG againstSARS-CoV-2 since symptom onset in 76 patients. (Blue: age < 65 year. Orange: age ≥ 65).

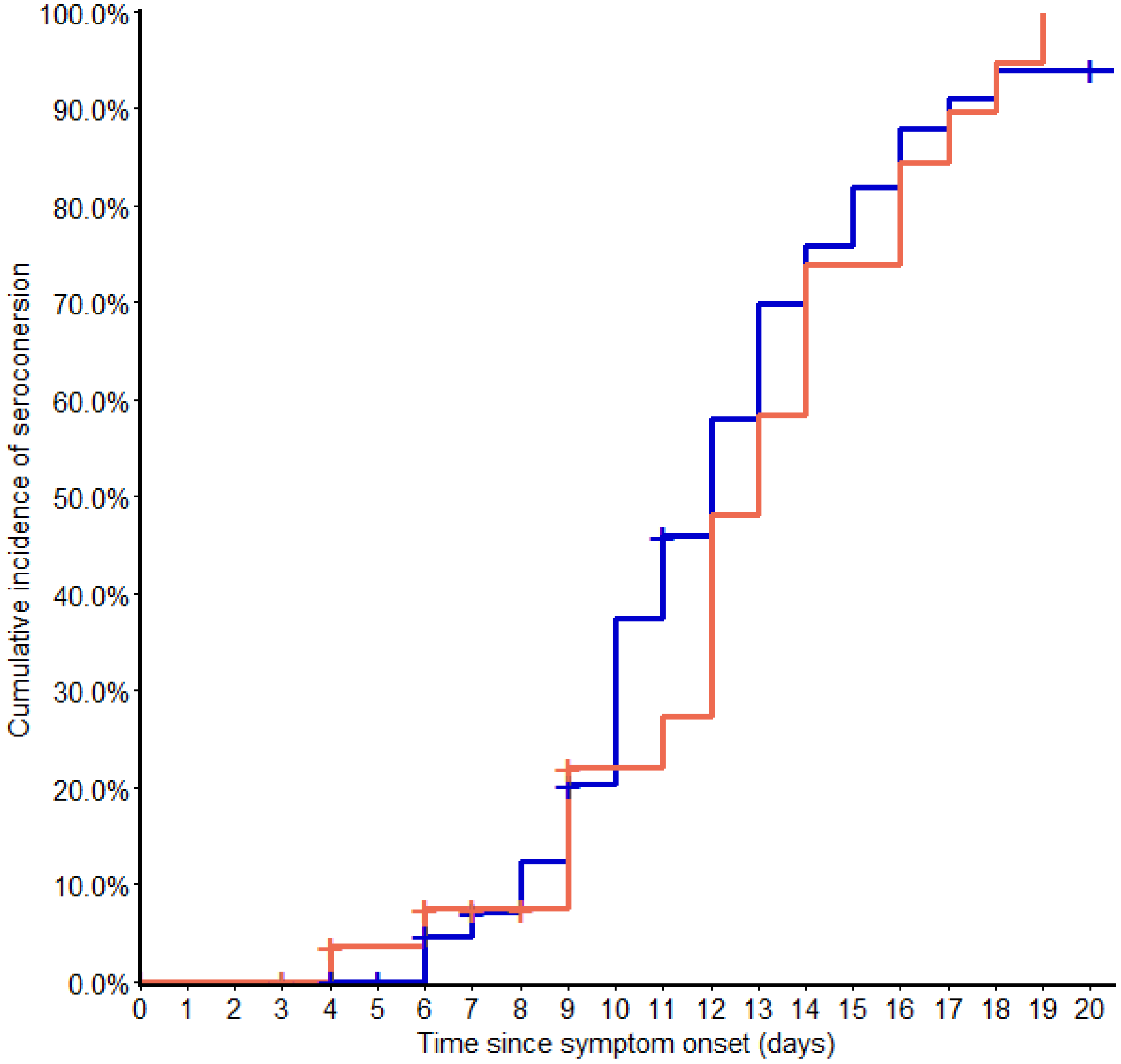

Fig. S7. Sensitivity analysis on fixed parameters.

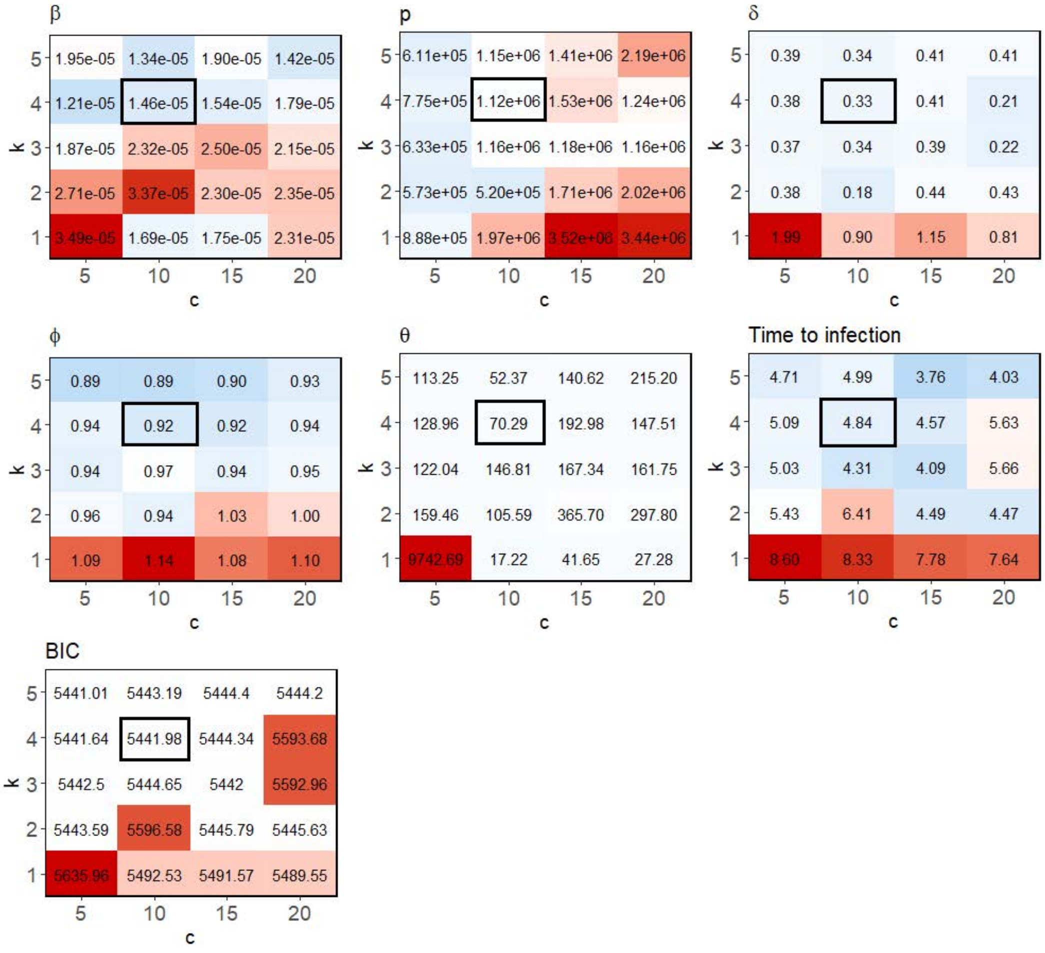

Estimation was performed with different value for each fixed parameter using the final model. BIC values and estimated population median values for $\beta, p, \delta, \ \phi, \theta$ and time of infection are reported. Colors indicate BIC or parameter values, with dark blue indicating lower values, white indicating median values and dark red indicating larger estimated values. The reference model is k=4 d^{-1} and c=10 d^{-1}(black border). The model fit with T_0=1.33×10^5 cells.mL^{-1}, d_f = 0.4 d^{-1}, μ=0.0001.

Fig. S8. Viral load at the time of death. Left: observed viral load at the time of death (+/- 2 days); Right: predicted viral load at the time of death using the final model

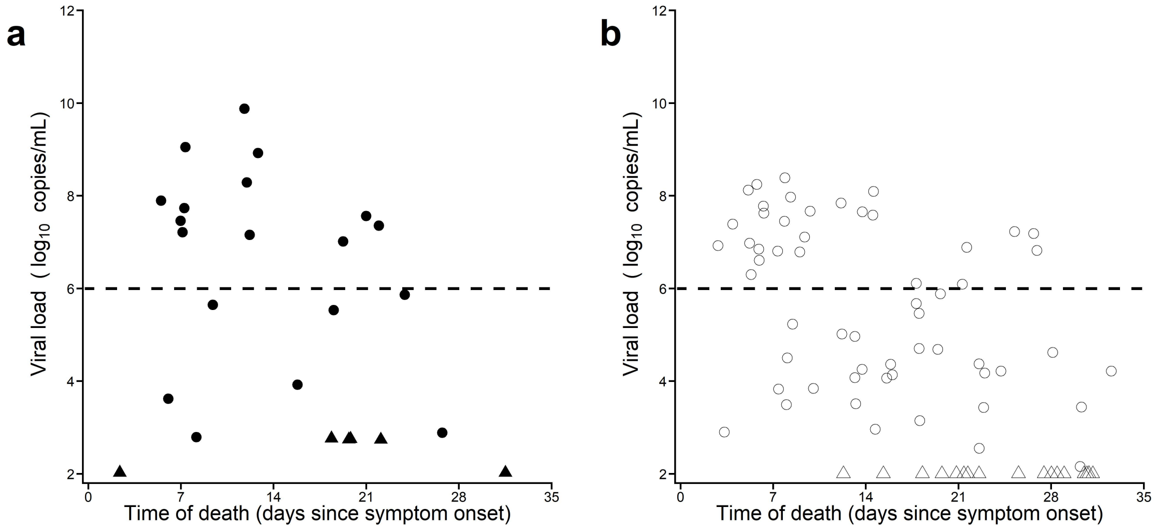

The dark circles and the black triangles represent the observed data and the data below the limit of detection, respectively. The empty circles and the empty triangles represent the viral load data predicted by the model at the time of death. Black horizontal dashed line represents threshold of positive culture.

Fig. S9. Visual predictive check of the predicted mortality.

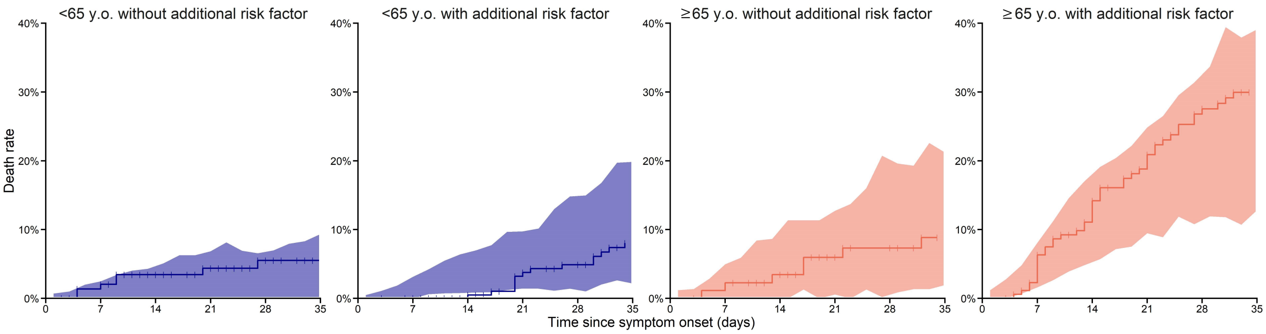

The shaded area are the 90% prediction interval using the technique proposed in Tardivon et al [2]. The solid line are the Kaplan-Meier estimates of survival in each population category.

Figure S10. a) Cumulative incidence of mortality in patients having at least one LRT sample (N=98, black) and those having no LRT samples (N=557, grey). Log-rank test (P < 0.0001) b) Correlation between NP and LRT viral load data in patients sampled on the same day (+/- 2 days).

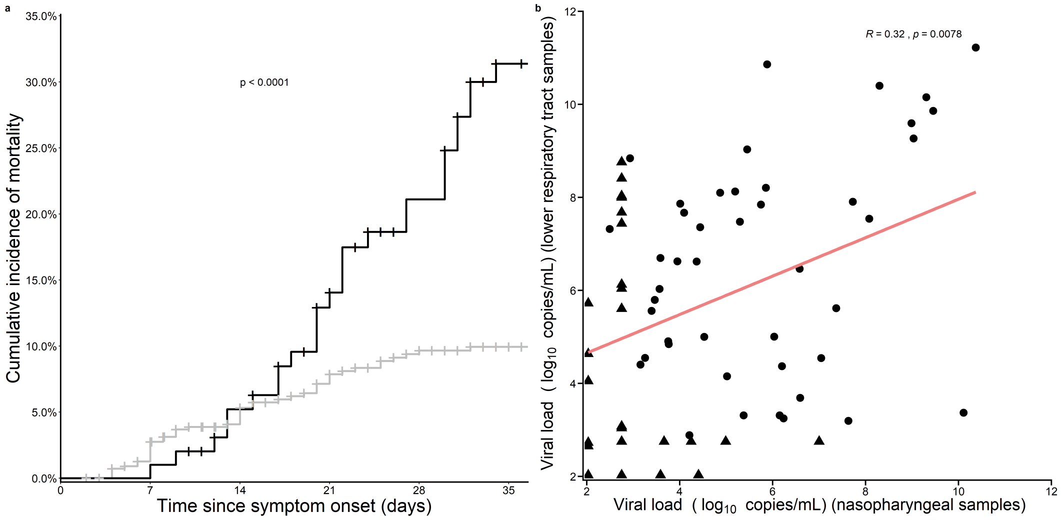

In patients having several pairs of data points, only the first one was used. Triangles are data below the limit of detection.

Table S1. Linear regression analysis on the factors associated with viral load at admission.

	Univariate analysis		Multivariate analysis	
**Parameter**	Coefficient	*P-value*	Coefficient	*P-value*
**Time since symptom onset**	-0.22	**<10^{-5}**	-0.21	**<10^{-5}**
**Age ≥65**	0.13	0.53		
**Male gender**	-0.16	0.45		
**Chronic cardiac disease**	0.6	**0.01**	0.62	**0.009**
**Chronic pulmonary disease**	0.15	0.65		

Table S2. Number of patients having nasopharyngeal (NP) samples or lower respiratory tract (LRT) samples according to the phase of the disease.

	Number of patients having NP samples			Number of patients having LRT samples		
**Data points available in each phase**	0-7 days since symptom onset N=326	7-14 days since symptom onset N=371	≥14 days since symptom onset N=222	0-7 days since symptom onset N=15	7-14 days since symptom onset N=46	≥14 days since symptom onset N=65
**1**	292 (89.6%)	289 (77.9%)	144 (64.9%)	13 (86.7%)	30 (65.2%)	21 (32.3%)
**2**	27 (8.3%)	58 (15.6%)	36 (16.2%)	1 (6.7%)	9 (19.6%)	19 (29.2%)
**3**	4 (1.2%)	20 (5.4%)	23 (10.4%)	1 (6.7%)	7 (15.2%)	15 (23.1%)
**≥4**	3 (0.9%)	4 (1.1%)	19 (8.5%)			

Table S3. Description of alternative models of immune response.

Models	Description of the model	Equation
**Model A: TCL model (without immune response)**	The model assumes a constant loss rate of infected cells	$$\frac{dI_2}{dt} = kI_1 - \delta I_2$$
**Model B: F leads target cells refractory to infection**	The model assumes a protection of target cells by rendering them definitely refractory to the infection	$$\frac{dT}{dt} = -\beta V_i T - \phi \frac{F}{F+\theta} T$$
**Model C: F decreases the rate of viral production**	The model assumes a decrease of non-infectious and infectious viruses' production in the same fashion	$$\frac{dV_i}{dt} = p \times \left(1 - \phi \frac{F}{F+\theta}\right) \times \mu I_2 - cV_i$$ $$\frac{dV_{ni}}{dt} = p \times \left(1 - \phi \frac{F}{F+\theta}\right) \times (1-\mu) I_2 - cV_{ni}$$
**Model D: F decreases infection rate**	The model assumes a protection of target cells by decreasing infection rate, decreasing the number of infected cells	$$\frac{dT}{dt} = -\beta \times \left(1 - \phi \frac{F}{F+\theta}\right) \times V_i T$$ $$\frac{dI_1}{dt} = \beta \times \left(1 - \phi \frac{F}{F+\theta}\right) \times V_i T - kI_1$$
**Model E: F increases viral clearance**	The model assumes two mechanisms of viral elimination	$$\frac{dV_i}{dt} = p\mu I_2 - cV_i - \phi \frac{F}{F+\theta} V_i$$
**Model F: F increases the clearance of infected cells**	The model assumes two mechanisms of cell elimination	$$\frac{dI_2}{dt} = kI_1 - \delta I_2 - \phi \frac{F}{F+\theta} I_2$$

Table S4. Selection procedure of longitudinal models tested.

Model	BIC	Residual error
**Model A**	4485.54	1.53
**Model B**	4504.24	1.54
**Model C**	4503.78	1.57
**Model D**	4488.10	1.54
**Model E**	4503.75	1.56
**Model F**	4487.59	1.45

The quality of model fit was evaluated by both the BIC and residual error for each of the models tested. The model providing both the lowest BIC and the lowest residual errors was retained.

Table S5. Description of alternative models of immune response that did not include an explicit antigen-dependent stimulation.

Models	Description of the model	Equation
**Model F with an age-dependent immune response**	The model assumes two mechanisms of cell elimination<br>The parameter φ is age-dependent	$\frac{dI_2}{dt} = kI_1 - \delta I_2 - \phi \frac{F}{F+\theta} I_2$<br>$\phi = \phi_0 + \phi_1 I_{\{age>65\}}$
**Model F1: TCL model with a time dependent adaptive immune response**	The model assumes a constant loss rate of infected cells<br>The parameter δ changes at time t=12 days post symptom onset to reflect antibody increase	$\frac{dI_2}{dt} = kI_1 - \delta I_2$<br>$\delta = \delta_0 + \delta_1 I_{\{time>12\}}$
**Model F2: TCL model with a time dependent adaptive immune response**	The model assumes a constant loss rate of infected cells<br>The parameter δ increases exponentially from t=12 days post symptom onset to t=30 days to reflect the progressive increase of antibody increase	$\frac{dI_2}{dt} = kI_1 - \delta I_2$<br>$\delta_\tau = \delta_0 + \delta_1\ exp^{w\times min((t-12),30)} I_{\{time>12\}}$
**Model F3: Assuming that F and I_2 are in quasi steady-state at all times ($\boldsymbol{F = a \times I_2}$) and that $\boldsymbol{\theta}$ >> $\boldsymbol{a \times I_2}$**	The model assumes two mechanisms of cell elimination, one linear and one quadratic<br>The quadratic term φ is age-dependent	$\frac{dI_2}{dt} = kI_1 - \delta I_2 - \phi I_2^2$<br>$\phi = \phi_0 + \phi_1 I_{\{age>65\}}$

Table S6. Parameter estimates of alternative models of immune response.

	Model F	Model F1	Model F2	Model F3
**Longitudinal model**	*Fixed effect (RSE%)*			
$\boldsymbol{\beta}$ **(mL.virus^{-1}. d^{-1})**	1.46×10^{-5} (23.4)	1.17×10^{-5} (21.2)	4.85×10^{-5} (16.7)	2.2×10^{-5} (> 100)
$\boldsymbol{pT_0}$ **(virus.mL^{-1}.d^{-1})**	1.48×10^{11} (26.8)	1.77×10^{11} (34.3)	5.71×10^{10} (31)	1.50×10^{11} (33)
$\boldsymbol{\delta}$ **(d^{-1})**	0.33 (30.0)	–	–	1.04 (6.5)
$\boldsymbol{\delta_2}$ **(d^{-1})**	–	0.13 (37.7)	–	–
$\boldsymbol{\delta_{age}(< 65)}$ **(d^{-1})**	–	1.23 (5.8)	1.04 (6.3)	–
$\boldsymbol{\delta_{age}(\geq 65)}$ **(d^{-1})**	–	0.98 (22.3)	0.82 (19.9)	–
$\boldsymbol{\omega}$ **(d^{-1})**	–	–	0.005 (52.3)	–
$\boldsymbol{\phi_{age}(< 65)}$ **(d^{-1})**	0.92 (8.67)	–	–	1.14×10^{-5} (14.8)
$\boldsymbol{\phi_{age}(\geq 65)}$ **(d^{-1})**	0.65 (23.3)	–	–	1.22×10^{-5}(572)
$\boldsymbol{\theta}$ **(F.mL^{-1})**	70 (80.8)	–	–	–
$\boldsymbol{T_{inf}}$ **(d)**	4.8 (3.2)	5.8 (8.5)	6.4 (6.0)	5.15 (8.6)
**Survival Model**	*Hazard Ratio (RSE%)*			
**Viral load (log$_{10}$ copies/mL)**	1.31 (17)	1.30 (16.1)	1.29 (19.3)	1.30 (15.8)
**Age ≥65**	2.58 (37.9)	2.58 (42.3)	2.58 (24.4)	2.83 (23.8)
**Male gender**	2.55 (25.2)	2.54 (33.1)	2.51 (42.9)	2.53 (25.6)
**Chronic pulmonary disease**	2.31 (36.8)	2.30 (30.7)	2.78 (46.5)	2.27 (32)
**BIC**	5441.98	5446.20	5457.06	5472.23

β: infection rate; δ: loss rate of infected cells; ω: exponential coefficient of increase immune response; p: rate of viral production; ϕ: maximal rate of immune cell clearance; θ: F concentration giving 50% of ϕ; T_{inf}: time to infection; T_0: uninfected target cells at baseline; σ: residual variability; RSE: relative standard error; BIC: Bayesian Information Criterion

Table S7. Survival analysis (using an exponential model for the baseline hazard function).

	Univariate analysis		Multivariate analysis	
**Parameter**	**Hazard ratio**	***P-value***	**Hazard ratio**	***P-value***
**Obesity**	1.29	0.31		
**Age ≥65**	3.22	$<10^{-5}$	3.02	$<10^{-5}$
**Male gender**	2.55	$<10^{-4}$	2.63	0.0003
**Chronic cardiac disease**	1.12	$<10^{-5}$		
**Chronic pulmonary disease**	3.12	$<10^{-4}$	2.47	0.0003

Table S8. Parameter estimates in the reference survival model and in survival models assuming a hazard starting at the day of infection, day of symptom onset, 7 and 10 days of infection.

	Reference model (RSE, %): with hazard starting at day of admission)		Model H1: with hazard starting at day of infection		Model H2: with hazard starting at day of symptom onset		Model H3: with hazard starting at day 7 of infection		Model H4: with hazard starting at day 10 of infection	
**Male gender**	2.55 (25.2)	<0.001	2.49 (22)	<0.001	2.48 (30.2)	<0.001	2.47 (129%)	>0.05	2.47 (33.6)	0.002
**Age ≥65**	2.58 (37.9)	<0.001	2.88 (28.5)	<0.001	2.80 (23.4)	<0.001	2.63 (32%)	0.002	2.47 (53.1)	>0.05
**Chronic pulmonary disease**	2.31 (36.8)	<0.001	2.25 (32.4)	0.002	2.23 (32)	0.002	2.24 (39.3%)	0.01	2.30 (35)	0.004
**Log_{10} viral load**	1.30 (17)	<0.001	1.05 (61.5)	0.10	1.11 (50.3)	0.04	1.21 (77%)	>0.05	1.35 (14.6)	<0.001
**-2LL**	5325.26		5408.54		5387.73		5361.95		5352.26	
**BIC**	5441.98		5525.26		5504.45		5478.68		5468.99	

List of authors of the French COVID Cohort Study groups

Name	*Last name*	*Affiliation*
Laurent	ABEL	Inserm UMR 1163, Paris, France
Claire	ANDREJAK	CHU Amiens, France
François	ANGOULVANT	Hôpital Necker, Paris, France
Delphine	BACHELET	Hôpital Bichat, Paris, France
Marie	BARTOLI	ANRS, Paris, France
Romain	BASMACI	Hôpital Louis Mourier, Colombes, France
Sylvie	BEHILILL	Pasteur Institute, Paris, France
Marine	BELUZE	F-CRIN Partners Platform, Paris, France
Dehbia	BENKERROU	Inserm UMR 1136, Paris, France
Krishna	BHAVSAR	Hôpital Bichat, Paris, France
François	BOMPART	Drugs for Neglected Diseases initiative, Geneva, Switzerland
Lila	BOUADMA	Hôpital Bichat, Paris, France
Maude	BOUSCAMBERT	Inserm UMR 1111, Lyon, France
Minerva	CERVANTES-GONZALEZ	REACTing, Paris, France
Anissa	CHAIR	Hôpital Bichat, Paris, France
Catherine	CHIROUZE	CHRU Jean Minjoz, Besançon, France
Alexandra	COELHO	Inserm UMR 1018, Paris, France
Sandrine	COUFFIN-CADIERGUES	Inserm sponsor, Paris, France
Camille	COUFFIGNAL	Hôpital Bichat, Paris, France
Eric	d’ORTENZIO	REACTing, Paris, France
Charlene	DA SILVEIRA	Hôpital Bichat, Paris, France
Marie-Pierre	DEBRAY	Hôpital Bichat, Paris, France
Dominique	DEPLANQUE	Hôpital Calmette, Lille, France
Diane	DESCAMPS	Hôpital Bichat, Paris, France
Mathilde	DESVALLÉE	Inserm UMR 1219, Bordeaux, France
Alpha	DIALLO	ANRS, Paris, France

Alphonsine	DIOUF	Inserm UMR 1018, Paris, France
Céline	DORIVAL	Inserm UMR 1136, Paris, France
François	DUBOS	CHU Lille, France
Xavier	DUVAL	Hôpital Bichat, Paris, France
Philippine	ELOY	Hôpital Bichat, Paris, France
Vincent	ENOUF	Pasteur Institute, Paris, France
Hélène	ESPEROU	Inserm sponsor, Paris, France
Marina	ESPOSITO-FARESE	Hôpital Bichat, Paris, France
Manuel	ETIENNE	CHU Rouen, France
Nadia	ETTALHAOUI	Hôpital Bichat, Paris, France
Nathalie	GAULT	Hôpital Bichat, Paris, France
Alexandre	GAYMARD	Inserm UMR 1111, Lyon, France
Jade	GHOSN	Hôpital Bichat, Paris, France
Tristan	GIGANTE	F-CRIN INI-CRCT, Nancy, France
Morgane	GILG	F-CRIN INI-CRCT, Nancy, France
Isabelle	GORENNE	Hôpital Bichat, Paris, France
Jérémie	GUEDJ	Inserm UMR 1137, Paris, France
Alexandre	HOCTIN	Inserm UMR 1018, Paris, France
Ikram	HOUAS	Inserm sponsor, Paris, France
Isabelle	HOFFMANN	Hôpital Bichat, Paris, France
Jean-Sébastien	HULOT	Hôpital Européen Georges Pompidou, Paris, France
Salma	JAAFOURA	Inserm sponsor, Paris, France
Ouifiya	KAFIF	Hôpital Bichat, Paris, France
Florentia	KAGUELIDOU	Hôpital Robert Debré, Paris, France
Sabrina	KALI	Hôpital Bichat, Paris, France
Antoine	KHALIL	Hôpital Bichat, Paris, France
Coralie	KHAN	Inserm UMR 1219, Bordeaux, France
Cédric	LAOUÉNAN	Hôpital Bichat, Paris, France
Samira	LARIBI	Hôpital Bichat, Paris, France
Minh	LE	Hôpital Bichat, Paris, France
Quentin	LE HINGRAT	Hôpital Bichat, Paris, France
Hervé	LE NAGARD	Inserm UMR 1137, Paris, France

Soizic	LE MESTRE	ANRS, Paris, France
François-Xavier	LESCURE	Hôpital Bichat, Paris, France
Yves	LEVY	Vaccine Research Insitute (VRI), Inserm UMR 955, Créteil, France
Claire	LEVY-MARCHAL	F-CRIN INI-CRCT, Paris, France
Bruno	LINA	Inserm UMR 1111, Lyon, France
Guillaume	LINGAS	Inserm UMR 1137, Paris, France
Jean Christophe	LUCET	Hôpital Bichat, Paris, France
Denis	MALVY	CHU Bordeaux, France
Marina	MAMBERT	Inserm UMR 1018, Paris, France
France	MENTRÉ	Hôpital Bichat, Paris, France
Noémie	MERCIER	ANRS, Paris, France
Amina	MEZIANE	Inserm UMR 1136, Paris, France
Hugo	MOUQUET	Pasteur Institute, Paris, France
Jimmy	Mullaert	Hôpital Bichat, Paris, France
Nadège	NEANT	Inserm UMR 1137, Paris, France
Marion	NORET	RENARCI, Annecy, France
Justine	PAGES	Hôpital Robert Debré, Paris, France
Aurélie	PAPADOPOULOS	Inserm sponsor, Paris, France
Christelle	PAUL	ANRS, Paris, France
Nathan	PEIFFER-SMADJA	Hôpital Bichat, Paris, France
Ventzislava	PETROV-SANCHEZ	ANRS, Paris, France
Gilles	PEYTAVIN	Hôpital Bichat, Paris, France
Olivier	PICONE	Hôpital Louis Mourier, Colombes, France
Oriane	PUÉCHAL	REACTing, Paris, France
Manuel	ROSA-CALATRAVA	Inserm UMR 1111, Lyon, France
Bénédicte	ROSSIGNOL	F-CRIN INI-CRCT, Nancy, France
Patrick	ROSSIGNOL	CHU Nancy, France
Carine	ROY	Hôpital Bichat, Paris, France
Marion	SCHNEIDER	Hôpital Bichat, Paris, France
Caroline	SEMAILLE	REACTing, Paris, France
Nassima	SI MOHAMMED	Hôpital Bichat, Paris, France
Lysa	TAGHERSET	Hôpital Bichat, Paris, France

Coralie	TARDIVON	Hôpital Bichat, Paris, France
Marie-Capucine	TELLIER	Hôpital Bichat, Paris, France
François	TÉOULÉ	Inserm UMR 1136, Paris, France
Olivier	TERRIER	Inserm UMR 1111, Lyon, France
Jean-François	TIMSIT	Hôpital Bichat, Paris, France
Théo	TRIOUX	Hôpital Bichat, Paris, France
Christelle	TUAL	Inserm CIC-1414, Rennes, France
Sarah	TUBIANA	Hôpital Bichat, Paris, France
Sylvie	VAN DER WERF	Pasteur Institute, Paris, France
Noémie	VANEL	Hôpital la Timone, Marseille, France
Aurélie	VEISLINGER	Inserm CIC-1414, Rennes, France
Benoit	VISSEAUX	Hôpital Bichat, Paris, France
Aurélie	WIEDEMANN	Vaccine Research Insitute (VRI), Inserm UMR 955, Créteil, France
Yazdan	YAZDANPANAH	Hôpital Bichat, Paris, France

List of authors of the French COVID Cohort Investigators group

Co-author 1		
Name	***Last name***	***Affiliation***
Raphael	BORIE	Paris - Bichat - SMIT
Etienne	DE MONTMOLLIN	Paris - Bichat - Réanimation
Duc	NGUYEN	Bordeaux - SMIT
Nicolas	BENECH	Lyon - SMIT
Elisabeth	BOTELHO-NEVERS	Saint Etienne - SMIT
Olivier	EPAULARD	Grenoble - SMIT
Camille	CHASSIN	Bourgoin-Jallieu - Médecine interne
Aldric	MANUEL	Annecy - SMIT
François Xavier	CATHERINE	Dijon - SMIT
Manuel	ETIENNE	Rouen - SMIT
Julien	POISSY	Lille - Réanimation
Eric	SENNEVILLE	Tourcoing - SMIT
Karine	FAURE	Lille - SMIT
Clotilde	ALLAVENA	Nantes - SMIT
Stéphane	SALLABERRY	Annecy - Réanimation
Elisa	DEMONCHY	Nice - SMIT
Fabrice	LAINE	Rennes - SMIT
Valentine	CAMPANA	Fort de France - SMIT
Julie	CHAS	Paris - Tenon -SMIT
Antoine	KIMMOUN	Nancy - Réanimation
François	GOEHRINGER	Nancy - SMIT
Erwan	L'HER	Brest - Réanimation
Vincent	LE MOING	Montpellier - SMIT
Alexa	DEBARD	Toulouse - SMIT
Paul	LOUBET	Nimes - SMIT
Hugues	CORDEL	Bobigny - Avicenne - SMIT
Laurent	BITKER	Lyon - Réanimation
Felix	DJOSSOU	Cayenne - SMIT/Réanimation
Vincent	DINOT	Metz - Réanimation
Rafael	MAHIEU	Angers - SMIT
Charline	VAUCHY	Besancon - SMIT
Martin	MARTINOT	Colmar - SMIT
Gwenhaël	COLIN	La Roche Sur Yon - Infectiologie

Denis	GAROT	Tours - Réanimation
Cécile	GOUJARD	Kremlin-Bicêtre -SMIT/Médecine interne
Isabelle	ENDERLE	Rennes - Gynécologie
Séverine	ANSART	Brest - SMIT
Guillermo	GIORDANO	Avignon - SMIT
Vincent	PEIGNE	Chambery - SMIT
Sylvain	DIAMANTIS	Melun - SMIT
Elodie	CURLIER	Guyane - Guadeloupe -Réanimation - SMIT
Jean-Daniel	LELIEVRE	Créteil - Mondor - SMIT
Nadia	SAIDANI	Quimper - MIIS
Victoria	MANDA	Paris - Lariboisière - SMIT
Adrien	LEMAIGNEN	Tours - SMIT
Cecile	AZOULAY	Paris - Cochin - CIC Vaccinologie
Laurent	LEFEBVRE	Aix en Provence - SMIT
Johann	AUCHABIE	Chollet - Réanimation
Roxane	COURTOIS	Chollet - SMIT
Karine	LACOMBE	Paris - Saint Antoine - SMIT
Nathalie	DE CASTRO	Paris - Saint Louis - Réanimation
Blandine	RAMMAERT	Poitiers - SMIT
Jean-Luc	DIEHL	Paris - HEGP - Réanimation
Hugues	AUMAÎTRE	Perpignan - SMIT
Grégory	CORVAISIER	Vannes - SMIT
Cédric	JOSEPH	Amiens - SMIT/Réanimation
Pierre-Adrien	BOLZE	Lyon Sud - Obstétrique
Firouzé	BANI-SADR	Reims - SMIT
Simon	BESSIS	Garches - SMIT
Hajnal-Gabriela	ILLES	Mont de Marsan - SMIT
Antoine	MERCKX	Cahors - SMIT
Younes	KERROUMI	Diaconesses CSS - Médecine interne
Cyril	LE BRIS	Beziers - SMIT/Réanimation
Brigitte	ELHARRAR	Créteil CHIC - Médecine interne
Nathalie	ALLOU	Saint Denis - Saint Pierre - SMIT
Corinne	DANIEL	Saint Martin - Médecine UDSMT
Cécile	FICKO	Bégin -SMIT
Benoît	ROZE	Saintes - Réanimation

Valérie	GABORIEAU	Pau - SMIT/Réanimation
Segolene	GREFFE	Boulogne Billancourt - A. Paré -Médecine interne
Olivier	LESENS	Clermont-Ferrand - SMIT
Jean-Charles	GAGNARD	Antony - Médecine interne
Simon-Djamel	THIBERVILLE	Manosque - SMIT
Stanislas	REBAUDET	Marseille - SMIT
Pauline	CARAUX PAZ	Villeneuve Saint Georges - SMIT
Moïse	MACHADO	Marne la Vallee- SMIT
Olivier	PICONE	Gynécologie,Hôpital Louis Mourrier, Colombe
Elsa	NYAMANKOLLY	Dax - SMIT/Réanimation
Jean-Benoit	ZABBE	Perigueux - SMIT
Camille	BOUISSE	Bourg en Bresse - Infectiologie/Réanimation
Ali	HACHEMI	Soissons - Infectiologie
François	BISSUEL	Thonon les Bains - Pneumologie
Mélanie	RORIZ	Agen - Médecine Interne
Anne Sophie	RESSEGUIER	Puy en Velay - Médecine interne
Marie	LACOSTE	Contamine sur Arve - Infectiologie/Réanimation
Anne Sophie	BOUREAU	Nantes - Gériatrie
Olivier	LAIREZ	Toulouse-cardiologie
Laurent	GUILLEMINAULT	Toulouse Larrey - Pneumologie
Marc	LAMBERT	Lille Calmette - SMIT
Hélène	SALVATOR	Suresnes - Hopital Foch - DRCI
Karen	DELAVIGNE	Toulouse - Hématologie/Médecine interne
Christophe	RAPP	Neuilly sur Seine - Médecine Interne
Eric	DELAVEUVE	Thionville - Bel Air - SMIT/Réanimation
Bertrand	DUSSOL	Marseille conception - Néphrologie
Marc	LEONE	Marseille Nord - La Timone - Réanimation
Jeanne	TRUONG	Paris - Robert Debré - Pédiatrie
Hikombo	HITOTO	Le Mans CH - SMIT
Vincent	LANGLOIS	Le Havre - MI / Pneumologie
Axelle	BRACONNIER	Mayotte - Gynécologie

Co-author 2		
Name	***Last Name***	***Affiliation***
Tiphaine	GOULENOK	Paris - Bichat - SMIT
Juliette	PATRIER	Paris - Bichat - Réanimation
Thomas	PERPOINT	Lyon - SMIT
Amandine	GAGNEUX-BRUNON	Saint Etienne - SMIT
Nicolas	TERZI	Grenoble - SMIT
Gabriel	MACHEDA	Annecy - SMIT
Mathieu	BLOT	Dijon - SMIT
Véronique	LEMEE	Rouen - SMIT
Saad	NSEIR	Lille - Réanimation
Olivier	ROBINEAU	Tourcoing - SMIT
Fanny	VUOTTO	Lille - SMIT
Sabelline	BOUCHEZ	Nantes - SMIT
Céline	MICHELANGELLI	Nice - SMIT
Matthieu	LESOUHAITIER	Rennes - SMIT
Jérémie	PASQUIER	Fort de France - SMIT
Bruno	LEVY	Nancy - Réanimation
Christian	RABAUD	Nancy - SMIT
Laetitia	BODENES	Brest - Réanimation
Nathalie	PANSU	Montpellier - SMIT
Pierre	DELOBEL	Toulouse - SMIT
Alberto	SOTTO	Nimes - SMIT
Nathalie	DOURNON	Bobigny - Avicenne - SMIT
Mehdi	MEZIDI	Lyon - Réanimation
Mayka	MERGEAYFABRE	Cayenne - SMIT/Réanimation
Rostane	GACI	Metz - Réanimation
Alexandra	DUCANCELLE	Angers - SMIT
Kévin	BOUILLER	Besancon - SMIT
Romain	DECOURS	La Roche Sur Yon - Infectiologie
Laurent	PLANTIER	Tours - Réanimation
Stéphane	JAUREGUIBERRY	Kremlin-Bicêtre -SMIT/Médecine interne
Cécile	TROMEUR	Brest - SMIT
Clara	MOUTON PERROT	Avignon - SMIT
Carola	PIEROBON	Chambery - SMIT
Catherine	CHAKVEATZE	Melun - SMIT
Rachida	OUISSA	Guyane - Guadeloupe -Réanimation - SMIT

Laurène	AZEMAR	Paris - Lariboisière - SMIT
Julie	MANKIKIAN	Tours - SMIT
Nicolas	CARLIER	Paris - Cochin - CIC Vaccinologie
Pascal	GRANIER	Aix en Provence - SMIT
Anthony	LEMEUR	Chollet - Réanimation
Thibault	CHIARABINI	Paris - Saint Antoine - SMIT
Geoffrey	LIEGEON	Paris - Saint Louis - Réanimation
Gwenaël	Le Moal	Poitiers - SMIT
Marine	LIVROZET	Paris - HEGP - Réanimation
Delphine	LARIVIERE	Vannes - SMIT
Julien	MOYET	Amiens - SMIT/Réanimation
Maxime	HENTZIEN	Reims - SMIT
Bouchra	LOUTFI	Mont de Marsan - SMIT
Vanina	MEYSSONNIER	Diaconesses CSS - Médecine interne
Benoit	THILL	Beziers - SMIT/Réanimation
Valerie	GARRAIT	Créteil CHIC - Médecine interne
Marie	LAGRANGE	Saint Denis - Saint Pierre - SMIT
Marie	GOMINET	Bégin -SMIT
Delphine	BREGEAUD	Saintes - Réanimation
Eve	LE COUSTUMIER	Pau - SMIT/Réanimation
Frédérique	RETORNAZ	Marseille - SMIT
Laurent	RICHIER	Villeneuve Saint Georges - SMIT
Audrey	BARRELET	Marne la Vallee- SMIT
Jeanne	SIBIUDE	Colombes - Louis Mourier - Gynécologie
Adrien	AUVET	Dax - SMIT/Réanimation
Florent	PEELMAN	Perigueux - SMIT
Nicholas	SEDILLOT	Bourg en Bresse - Infectiologie/Réanimation
Patrick	RISPAL	Agen - Médecine Interne
Marlène	MURRIS	Toulouse Larrey - Pneumologie
Arnaud	SCHERPEREEL	Lille Calmette - SMIT
Erwan	FOURN	Suresnes - Hopital Foch - DRCI
Stéphane	LASRY	Neuilly sur Seine - Médecine Interne
Coline	JAUD-FISCHER	Thionville - Bel Air - SMIT/Réanimation
Bruno	PASTENE	Marseille Nord - La Timone - Réanimation
Laure	GOUBERT	Le Havre - MI / Pneumologie

Co-author 3		
Name	***Last name***	***Affiliation***
Dominique	LUTON	Paris - Bichat - SMIT
Paul Henri	WICKY	Paris - Bichat - Réanimation
Anne	CONRAD	Lyon - SMIT
Tiffany	TROUILLON	Saint Etienne - SMIT
Jean-François	PAYEN	Grenoble - SMIT
Mylène	MAILLET	Annecy - SMIT
Sophie	MAHY	Dijon - SMIT
Eglantine	FERRAND DEVOUGE	Rouen - SMIT
Sébastien	PREAU	Lille - Réanimation
Agnès	MEYBECK	Tourcoing - SMIT
Marie-Charlotte	CHOPIN	Lille - SMIT
Romain	GUERY	Nantes - SMIT
Karine	RISSO	Nice - SMIT
Matthieu	REVEST	Rennes - SMIT
André	CABIE	Fort de France - SMIT
Maximilen	SAINT GILLES	Nancy - Réanimation
Sibylle	BEVILACQUA	Nancy - SMIT
Nicolas	FERRIERE	Brest - Réanimation
Clément	LE BIHAN	Montpellier - SMIT
Benjamine	SARTON	Toulouse - SMIT
Didier	LAUREILLARD	Nimes - SMIT
Olivier	BOUCHAUD	Bobigny - Avicenne - SMIT
Hodane	YONIS	Lyon - Réanimation
Arsène	KPANGON	Cayenne - SMIT/Réanimation
Nadia	OUAMARA	Metz - Réanimation
Vincent	DUBEE	Angers - SMIT
Maïder	PAGADOY	Besancon - SMIT
Thomas	GUIMARD	La Roche Sur Yon - Infectiologie
Valérie	GISSOT	Tours - Réanimation
Antoine	CHERET	Kremlin-Bicêtre -SMIT/Médecine interne
Dewi	GUELLEC	Brest - SMIT
Vincent	PESTRE	Avignon - SMIT
Marie-Christine	CARRET	Chambery - SMIT
Clara	FLATEAU	Melun - SMIT
Isabelle	FABRE	Guyane - Guadeloupe -Réanimation - SMIT
Guylaine	CASTOR-ALEXANDRE	Paris - Lariboisière - SMIT
Thomas	FLAMENT	Tours - SMIT
Liem	LUONG	Paris - Cochin - CIC Vaccinologie

Laurence	MAULIN	Aix en Provence - SMIT
Thierry	MAZZONI	Chollet - Réanimation
Bénédicte	LEFEBVRE	Paris - Saint Antoine - SMIT
Diane	PONSCARME	Paris - Saint Louis - Réanimation
Isabelle	PIRONNEAU	Poitiers - SMIT
Bernard	CHOLLEY	Paris - HEGP - Réanimation
Marie	LANGELOT-RICHARD	Vannes - SMIT
Cinthia	RAMES	Amiens - SMIT/Réanimation
Yohan	N'GUYEN	Reims - SMIT
Jérôme	DIMET	Mont de Marsan - SMIT
Oryane	MABIALA	Diaconesses CSS - Médecine interne
Marie-Laure	CASANOVA	Beziers - SMIT/Réanimation
Isabelle	DELACROIX	Créteil CHIC - Médecine interne
Julien	JABOT	Saint Denis - Saint Pierre - SMIT
Aurore	BOUSQUET	Bégin -SMIT
Younes	AIT TAMLIHAT	Saintes - Réanimation
Walter	PICARD	Pau - SMIT/Réanimation
Myriam / Hortense	BENNANI / DROUET	Marseille - SMIT
Danielle	JAAFAR	Villeneuve Saint Georges - SMIT
Alexandra	BEDOSSA	Marne la Vallee- SMIT
Laurent	MANDELBROT	Colombes - Louis Mourier - Gynécologie
Anne-Hélène	BOIVIN	Dax - SMIT/Réanimation
Edouard	SOUM	Perigueux - SMIT
Damien	BOUHOUR	Bourg en Bresse - Infectiologie/Réanimation
Sarah	REDL	Agen - Médecine Interne
Agnès	SOMMET	Toulouse Larrey - Pneumologie
Ryadh	POKEERBUX	Lille Calmette - SMIT
David	ZUCMAN	Suresnes - Hopital Foch - DRCI
Thierry	CARMOI	Neuilly sur Seine - Médecine Interne
Paul	DUNAND	Thionville - Bel Air - SMIT/Réanimation
Karine	BEZULIER	Marseille Nord - La Timone - Réanimation
Stéphanie	COUSSE	Le Havre - MI / Pneumologie

Co-author 4		
Name	***Last name***	***Affiliation***
Lauren	DECONINCK	Paris - Bichat - SMIT
Lucie	LE FEVRE	Paris - Bichat - Réanimation
Laurence	BOUILLET	Grenoble - SMIT
Patrick	IMBERT	Annecy - SMIT
Marielle	BUISSON	Dijon - SMIT
Kévin	ALEXANDRE	Rouen - SMIT
Mercé	JOURDAIN	Lille - Réanimation
Sarah	STABLER	Lille - SMIT
Paul	LE TURNIER	Nantes - SMIT
Pierre	TATTEVIN	Rennes - SMIT
Pierre-François	SANDRINE	Fort de France - SMIT
Benjamin	LEFEVRE	Nancy - SMIT
Stella	ROUSSET	Toulouse - SMIT
Guillaume	LOUIS	Metz - Réanimation
Quentin	LEPILLER	Besancon - SMIT
Emmanuelle	MERCIER	Tours - Réanimation
Florence	JEGO	Chambery - SMIT
Pierre-Marie	ROGER	Guyane - Guadeloupe -Réanimation - SMIT
Marie	LACHATRE	Paris - Cochin - CIC Vaccinologie
Jean-Benoit	ARLET	Paris - HEGP - Réanimation
Juliette	ROMARU	Reims - SMIT
Georges	LE FALHER	Beziers - SMIT/Réanimation
Thomas	MAITRE	Créteil CHIC - Médecine interne
Claudine	BADR	Villeneuve Saint Georges - SMIT
Stéphanie	FRY	Lille Calmette - SMIT
Marie-Laure	CHABI-CHAVILLAT	Suresnes - Hopital Foch - DRCI

Co-author 5		
Name	***Last name***	***Affiliation***
Sylvie	LE GAC	Paris - Bichat - SMIT
Pierre	JACQUET	Paris - Bichat - Réanimation
Rebecca<br>Marion	HAMIDFAR<br>LE MARECHAL	Grenoble - SMIT<br>Grenoble - SMIT
Amélie	VALRAN	Annecy - SMIT
Lionel	PIROTH	Dijon - SMIT
Elise	ARTAUD-<br>MACCARI	Rouen - SMIT
Raphaël	FAVORY	- Réanimation
Jules	BAUER	Lille - SMIT
Cécile	MEAR-PASSARD	Nantes - SMIT
Jean-Marc	CHAPPLAIN	Rennes - SMIT
Jean-Marie	TURMEL	Fort de France - SMIT
Anne	GUILLAUMOT	Nancy - SMIT
Guillaume	MARTIN-<br>BLONDEL	Toulouse - SMIT
Cyril	CADOZ	Metz - Réanimation
Noémie	TISSOT	Besancon - SMIT
Charlotte	SALMON<br>GANDONNIERE	Tours - Réanimation
Margaux	ISNARD	Chambery - SMIT
Samuel	MARKOWICZ	Guyane - Guadeloupe -Réanimation - SMIT
Odile	LAUNAY	Paris - Cochin - CIC Vaccinologie
Olivier	SANCHEZ	Paris - HEGP - Réanimation
Kévin	DIDIER	Reims - SMIT
Eric	OZIOL	Beziers - SMIT/Réanimation
Jean Baptiste	ASSIE	Créteil CHIC - Médecine interne
Fara	DIOP	Villeneuve Saint Georges - SMIT
Cécile	YELNIK	Lille Calmette - SMIT
Aurélie	MARTIN	Suresnes - Hopital Foch - DRCI

References

[1] P. Institut Pasteur, Protocol: Real-time RT-PCR assays for the detection of SARS CoV-2. 569 (2020) (April 24, 2020).

[2] C. Tardivon, *et al.*, Association Between Tumor Size Kinetics and Survival in Patients With Urothelial Carcinoma Treated With Atezolizumab: Implication for Patient Follow-Up. *Clin. Pharmacol. Ther.* **106**, 810–820 (2019).
